# Alternative splicing of a species- and skeletal muscle-specific microexon diversifies the menu of clathrin heavy chains in mice and rats

**DOI:** 10.1016/j.isci.2026.117799

**Published:** 2026-10-07

**Authors:** R. Eric Blue, Adam J. Black, Jessica L. Cote, Sumaya A. Addish, Archishma Kavalipati, Scott P. Lyons, J. Ashley Ezzell, Steven C. Bremmer, Daniel Dominguez, Jimena Giudice

**Affiliations:** 1 Department of Cell Biology and Physiology, The University of North Carolina at Chapel Hill, Chapel Hill, NC 27599, USA; 2 Curriculum in Cell Biology and Physiology, The University of North Carolina at Chapel Hill, Chapel Hill, NC 27599, USA; 3 Department of Pharmacology, The University of North Carolina at Chapel Hill, Chapel Hill, NC 27599, USA; 4 Curriculum in Bioinformatics and Computational Biology, The University of North Carolina at Chapel Hill, Chapel Hill, NC 27599, USA; 5 UNC Metabolomics and Proteomics Core, The University of North Carolina at Chapel Hill, Chapel Hill, NC 27599, USA; 6 Lineberger Comprehensive Cancer Center, The University of North Carolina at Chapel Hill, Chapel Hill, NC 27599, USA; 7 RNA Discovery Center, The University of North Carolina at Chapel Hill, Chapel Hill, NC 27599, USA; 8 McAllister Heart Institute, The University of North Carolina at Chapel Hill, Chapel Hill, NC 27599, USA

**Keywords:** alternative splicing, microexons, RNA, clathrin, CLTC, CLTCL1, skeletal muscle, tissue specific

## Abstract

Little is known about alternative splicing of microexons in most tissues other than the brain. Humans have two homologous clathrin heavy chains. Ubiquitously expressed, CLTC drives clathrin-mediated endocytosis. Highly expressed in skeletal muscles, CLTCL1 instead regulates glucose metabolism. Previously, we reported that a conserved microexon (exon 31) in the *CLTC* gene is alternatively spliced during striated muscle development. Here, we identified a novel *Cltc* microexon (exon 33, hereafter microexon 33), which is only included in mouse and rat adult skeletal muscles. Microexon 33 inclusion shortens the CLTC C-terminus and adds 10 distinct terminal residues. This divergence occurs at the same position at which the human CLTCL1 constitutively includes its penultimate exon (another microexon). *CLTCL1* is a pseudogene in mice and rats. Humans diversified the menu of clathrin heavy chains by gene duplication and tissue-specific gene expression, whereas mice and rats might have achieved similar diversification by tissue-specific regulation of *Cltc* alternative splicing.

## Highlights

• *Cltc* microexon 33 alternative splicing is tissue and developmental stage specific
• *Cltc* microexon 33 appears to be specific for mouse and rat
• The inclusion of the unannotated microexon 33 truncates the CLTC C terminus
• Microexon 33 produces a CLTC C terminus ending with a distinct ATISASSASI peptide

## Graphical abstract

**Figure:**
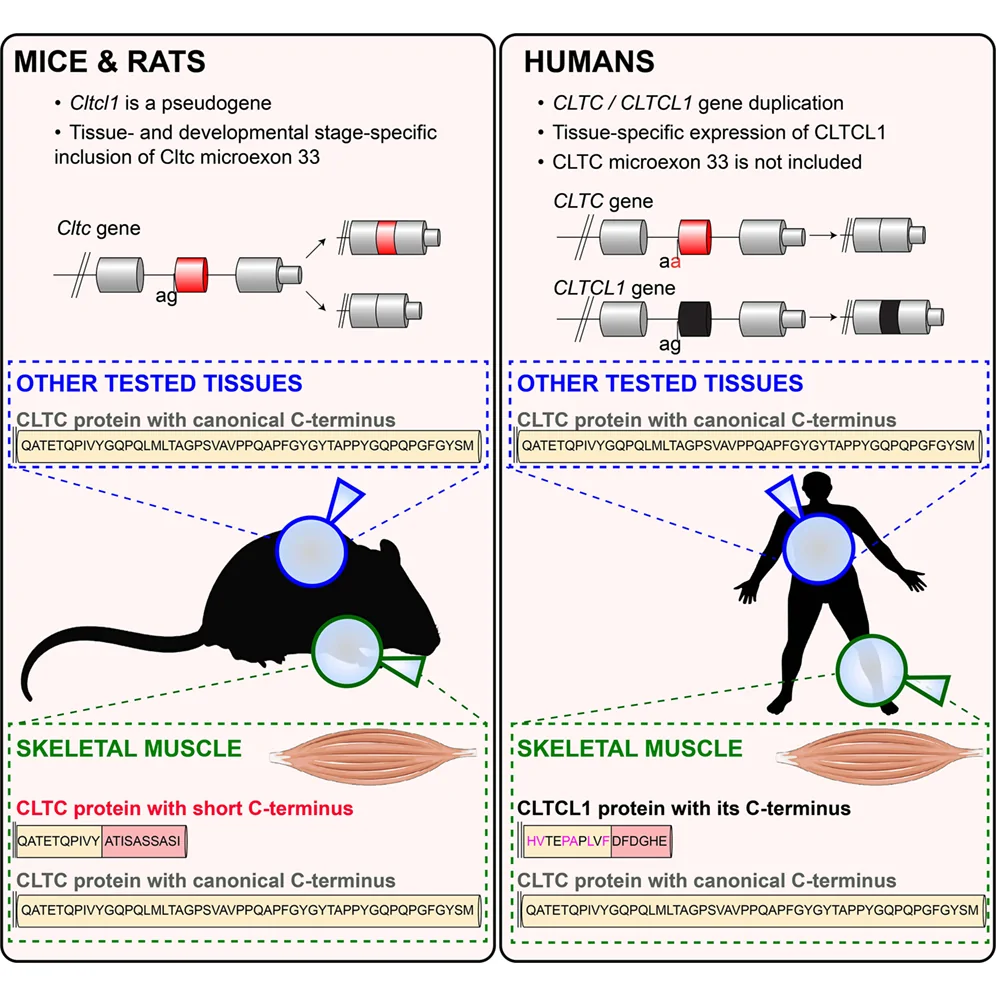

## Teaser

Molecular biology; Cell biology; Transcriptomics

## Introduction

Alternative splicing is an RNA-processing mechanism that allows individual genes to produce distinct transcripts. Alternative splicing is associated with tissue-identity acquisition and organ function during development and is very prevalent in humans, where ∼95% of multi-exonic genes are alternatively spliced.^1^^,^^2^^,^^3^^,^^4^^,^^5^^,^^6^ While most alternative splicing events observed in humans are not evolutionarily conserved, alternatively spliced exons that are tissue specific and/or regulated during periods of intense physiological change (e.g., in disease or development) show high conservation in their sequences and timing of their splicing transitions.^7^^,^^8^^,^^9^ The highest levels of tissue-specific and conserved alternative splicing have been detected in the heart, brain, and skeletal muscle.^2^^,^^10^

Very small exons, known as microexons, are often alternatively spliced in a tissue-specific and evolutionarily conserved manner. Microexon definition is based on exon length and varies between ≤51 nucleotides (nt),^11^^,^^12^^,^^13^ 3–27 nt,^14^ and 3–30 nt.^15^ In humans, 15,688 microexons have been identified,^16^ and >60% of them show high sequence conservation.^14^ Importantly, ∼80% of all alternatively spliced microexons have coding potential.^14^ Microexons are recognized as “tiny but mighty”^17^ because, despite their small size, they can make major contributions to protein function.^15^^,^^18^^,^^19^ Microexons with coding potential tend to encode peptides situated within intrinsically disordered regions of proteins.^14^ Alternative splicing of intrinsically disordered regions rewires tissue-specific protein-protein interaction networks^20^^,^^21^; coincidentally, alternative splicing of microexons tends to be tissue specific. The existence of alternatively spliced microexons and their regulation have been greatly investigated in brain development and autism spectrum disorders,^12^^,^^14^^,^^17^^,^^19^^,^^22^^,^^23^^,^^24^ but very little is known about alternative splicing of microexons in other tissues. During our studies in mouse skeletal muscle and heart development,^4^^,^^5^^,^^25^^,^^26^^,^^27^ we noticed that numerous alternatively spliced exons fall within the microexon category.

Here, we focus on the clathrin heavy chain (*Cltc*) gene. CLTC is an essential protein due to its starring role in clathrin-mediated endocytosis, which is a major endocytic route in mammals.^28^^,^^29^ CLTC is ubiquitously expressed and highly intolerant to missense or null variants: to date, only 35 human *CLTC* variants have been published, with many of them causing severe developmental conditions.^30^^,^^31^^,^^32^^,^^33^^,^^34^^,^^35^^,^^36^^,^^37^^,^^38^^,^^39^^,^^40^^,^^41^ CLTC is required for the formation of the skeletal muscle contractile apparatus and maximum muscle strength.^42^ These muscle-specific functions of CLTC are likely due, at least in part, to mechanisms that are distinct from clathrin-mediated endocytosis and might be related to its capacity to interact with and regulate the actin cytoskeleton.^42^^,^^43^^,^^44^^,^^45^^,^^46^ We previously reported that a 21-nt-long microexon (exon 31) in *Cltc* pre-mRNA is alternatively spliced during mouse heart development.^4^^,^^25^ This exon is herein referred to as *Cltc* microexon 31. We and others have since shown that Cltc microexon 31 is also developmentally regulated in skeletal muscle^5^^,^^9^^,^^25^^,^^26^^,^^47^ and influences skeletal muscle mass, intracellular architecture,^25^^,^^47^ and clathrin plaque formation.^47^ However, despite a robust body of literature surrounding the *Cltc* gene, some aspects of its structure and function have not yet been defined.

Humans and most other vertebrates, rodents being a significant exception, have two genes that encode highly homologous clathrin heavy chains: the *CLTC* gene (also known as CHC17 due to its location on chromosome 17) and the clathrin heavy chain-like 1 (*CLTCL1*) gene (also known as CHC22 due to its location on chromosome 22). The latter was generated by gene duplication during vertebrate evolution.^48^ CLTC and CLTCL1 protein sequences are 85% identical^48^ and both homotrimerize into clathrin triskelia that assemble to form latticed vesicle coats.^49^ However, CLTC and CLTCL1 differ in four ways. First, the CLTCL1 coat is more stable than the CLTC coat.^49^ Second, CLTC binds clathrin light chains while CLTCL1 does not.^49^^,^^50^ Third, in humans, CLTC is ubiquitously expressed whereas CLTCL1 is very highly expressed in skeletal muscle, lowly expressed in adipose tissue, and absent in all other tissues where it was studied thus far.^51^ Fourth, CLTC and CLTCL1 have been reported to perform distinct, non-compensatory roles.^51^^,^^52^^,^^53^^,^^54^^,^^55^ Whereas CLTC is a major contributor to clathrin-mediated endocytosis, CLTCL1 is not and can only substitute functionally for CLTC in clathrin-mediated endocytosis when expressed at extremely high, non-physiological levels and in the absence of endogenous CLTC.^51^^,^^52^^,^^53^^,^^54^^,^^55^ Furthermore, CLTCL1 regulates the targeting of the glucose transporter 4 (GLUT4) to the intracellular storage compartment until insulin triggers its translocation to the cell surface but CLTC does not.^50^^,^^56^^,^^57^ This CLTCL1-driven, insulin-responsive GLUT4 trafficking is the dominant mechanism in humans for clearing blood glucose into the skeletal muscle and adipose tissues following a meal.^58^^,^^59^ Perplexingly, mice and rats maintain a vital, insulin-responsive GLUT4 trafficking axis for clearing blood glucose even though in these species the *CLTCL1* gene has evolved as a pseudogene.^48^^,^^57^ This raises the following question: which other mechanisms have evolved in rodents to diversify the menu of clathrin heavy chain proteins and compensate for the loss of the *CLTCL1* gene?

Almost everything that we know about CLTC arises from studies of its canonical isoform, which lacks microexon 31 (21 nt in length) as well as another microexon (exon 33; 38 nt in length) that is currently unannotated and that we are reporting here. We herein refer to this exon as microexon 33. We show that while the sequence of microexon 31 is highly conserved during evolution, the sequence of microexon 33 is only conserved between mice and rats. Moreover, whereas microexon 31 is included in mouse heart, skeletal muscle, and brain, microexon 33 is only included in skeletal muscle and not in any of the other tested tissues. Thus, *Cltc* microexons 31 and 33 show striking differences in their tissue specificity and evolutionary conservation. In mouse and rat skeletal muscle, *Cltc* microexon 33 is mostly skipped in fetal and neonatal stages and is gradually included after birth, reaching a plateau after postnatal day 90 (P90). Microexon 33 is the penultimate exon in *Cltc* pre-mRNA; although it encodes a premature stop/termination codon (PTC), transcripts including it escape nonsense-mediated decay (NMD). Analysis of the sequence of microexon 33 and targeted mass-spectrometry studies revealed that inclusion of microexon 33 produces a shorter, distinct CLTC C-terminus where the last 41 amino acids of the canonical variant are replaced by a distinct 10-amino acid sequence (ATISASSASI). This divergence, specific to adult skeletal muscle in mouse and rat, occurs at the same residue position at which the other clathrin heavy chain protein expressed in humans (encoded by the *CLTCL1* gene) constitutively includes its penultimate exon, which interestingly is another microexon (40 nt in length). Therefore, whereas humans have diversified their menu of clathrin heavy chains by gene duplication (*CLTC* and *CLTCL1*) and differential tissue-specific expression of these genes, mice and rats may have achieved this diversification by tissue-specific regulation of the *Cltc* gene via alternative splicing.

## Results

### An unannotated microexon in *Cltc* pre-mRNA is regulated by alternative splicing during mouse skeletal muscle development

We have previously reported via RNA-sequencing studies that *Cltc* microexon 31 is alternatively spliced in cardiomyocytes during postnatal mouse development^4^ (Figure 1A, vertical blue box). *Cltc* microexon 31 is lowly included in neonatal cardiomyocytes (P1–3) (Figure 1A, first lane—purple tracks) and more included in adult (P60–67) cardiomyocytes^4^ (Figure 1A, second lane—green tracks). We and others^9^^,^^25^^,^^26^ have then shown that *Cltc* microexon 31 inclusion increases during skeletal muscle development (Figure 1A, third and fourth lanes). It is clear that *Cltc* microexon 31 alternative splicing is regulated to a larger extent during the development of skeletal muscles compared to that of cardiomyocytes. Importantly, microexon 31 is in-frame and encodes seven amino acids (VDAIKEK) located within the C-terminal region of the canonical CLTC protein isoform.

**Figure 1:**
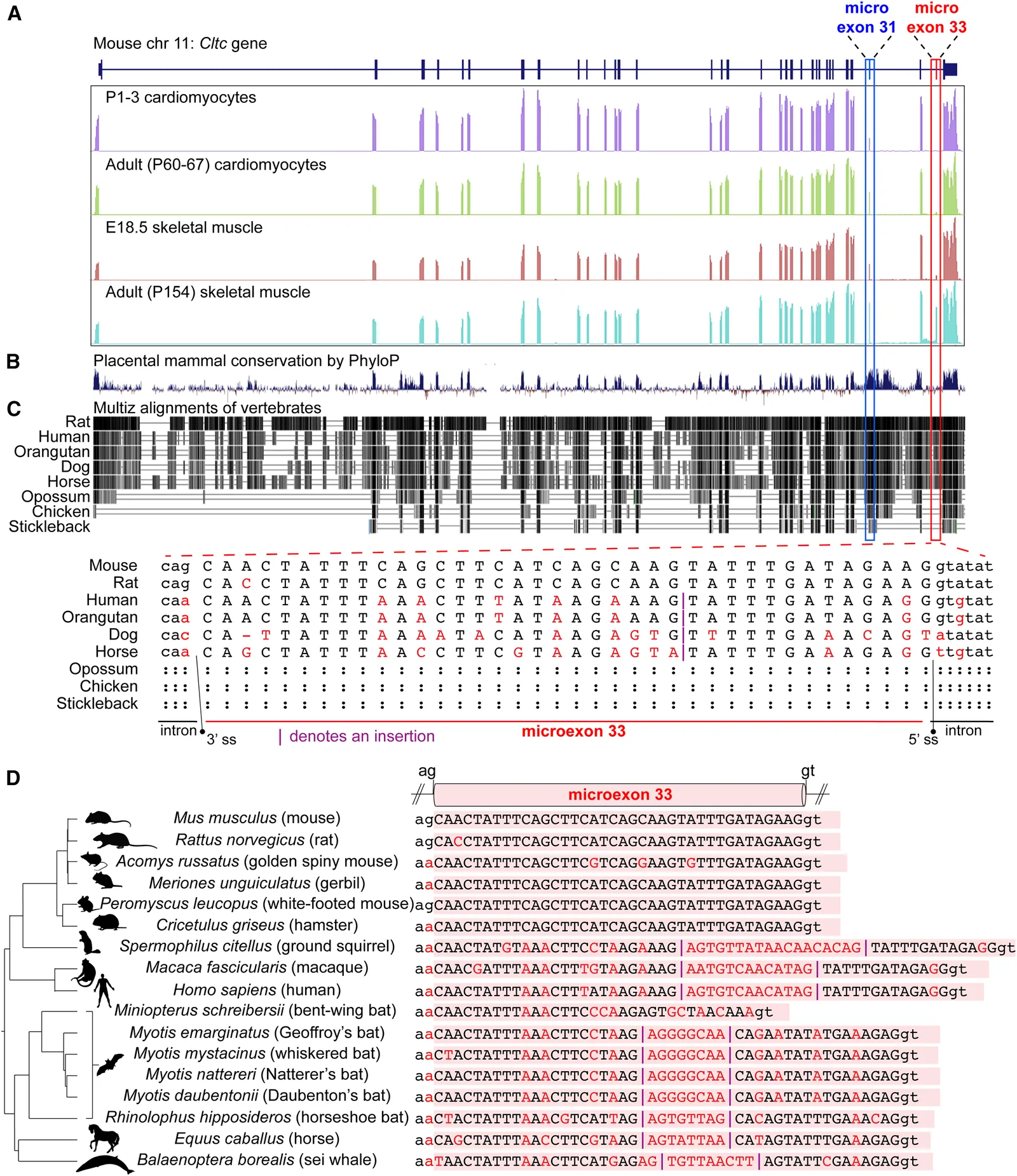
An unannotated and non-conserved microexon in *Cltc* pre-mRNA is regulated by alternative splicing during mouse skeletal muscle development (A) RNA-sequencing tracks from datasets in previously published studies in mouse cardiomyocyte development^4^ and skeletal muscle development^9^ showed two alternatively spliced microexons in the *Cltc* gene locus: microexon 31 (blue box) and microexon 33 (red box). TOPO cloning of *Cltc* microexon 33 confirms its sequence and size and is presented in Figure S1. (B) Plot of basewise evolutionary conservation of the *Cltc* gene as determined by PhyloP score and visualized in the UCSC Genome Browser. (C) Alignments of microexon 33 in different vertebrate species were obtained from the UCSC Genome Browser which generated them with multiz and other tools in the UCSC/Penn State Bioinformatics comparative genomics alignment pipeline. Bases that differ from those in the mouse sequence are shown in red. The purple vertical bars denote sequence insertions. Similar analysis for microexon 31 is presented in Figure S2. (D) Sequence alignment homology of the mouse *Cltc* microexon 33 versus other species. The mouse DNA sequence between *Cltc* exons 32 and 34 was imported into the NCBI BLAST nucleotide search tool. BLAST identified species that contained significant alignment to the mouse sequence input. The displayed sequences denote the individual nucleotides present in microexon 33 and its splice sites in different species. Bases that differ from the mouse sequence are shown in red. The purple brackets denote a sequence insertion. Species silhouettes were obtained from PhyloPic open database under CC0 1.0 Universal Public Domain Dedication. chr, chromosome; E18.5, embryonic day 18.5; P1–3, postnatal day 1 to 3 (i.e., samples prepared from P1, P2, and P3 animals were pooled together); ss, splice site. Analysis of Cltc microexon 33 with available genomes across the rodential order is presented in Figure S3. Analysis of the human *CLTC* intron downstream exon 32 is presented in Figure S4.

While revisiting the tracks of RNA-sequencing data that we generated during mouse cardiomyocyte development,^4^ and the tracks from data published by others on samples collected during mouse skeletal muscle development,^9^ we noticed that *Cltc* pre-mRNA contains another microexon that is currently unannotated in the genome. This microexon is located after exon 32, and thus we refer to it as microexon 33 (Figure 1A, vertical red box). Unlike microexon 31, microexon 33 is absent in mouse P1–3 cardiomyocytes and nearly absent in adult cardiomyocytes (Figure 1A, first and second lanes); yet, it is dramatically more included in adult versus embryonic (embryonic day 18.5, E18.5) skeletal muscles (Figure 1A, third versus fourth lanes).

These data indicate that microexon 33 in the mouse *Cltc* gene is developmentally regulated by alternative splicing to a large degree in skeletal muscle but only to a nominal degree in the heart. This finding contrasts with *Cltc* microexon 31, which is regulated by alternative splicing during both heart and skeletal muscle development in the mouse.^9^^,^^25^^,^^26^

### TOPO cloning *Cltc* microexon 33 confirms its sequence and size

We amplified cDNA from adult (P120) mouse gastrocnemius muscle, cloned it into the pCR4-TOPO vector, and used it to transform competent cells. We selected six individual colonies for further culturing, purified the plasmid DNA, and analyzed it by Sanger sequencing using the universal M13 reverse (M13R) and M13 forward (M13F) primers (Figure S1, black arrows at the top). In five out of six colony preparations, we detected the presence of *Cltc* microexon 33 and confirmed that its sequence (CAA-CTA-TTT-CAG-CTT-CAT-CAG-CAA-GTA-TTT-GAT-AGA-AG) and size (38 nt) (Figure S1) were consistent with the observations that we made from the RNA-sequencing data.

### Microexon 33 and its surrounding intronic regions show low evolutionary conservation

We evaluated sequence conservation of *Cltc* microexons 31 and 33 using PhyloP scores and Multiz alignments, both of which are available in the UCSC (University of California Santa Cruz) Genome Browser. Whereas *Cltc* microexon 31 and its surrounding intronic regions were highly evolutionarily conserved, microexon 33 and its surrounding intronic regions exhibited very low conservation (Figure 1B). Indeed, every single nucleotide of microexon 31 as well as its 3′ and 5′ splice sites were fully conserved in mouse, rat, human, orangutan, dog, horse, opossum, and chicken (Figure S2). In contrast, while the sequences of microexon 33 and its splice sites were identical in mice and rats except for one nucleotide (an A-to-C change in rat), several nucleotide differences and insertions were detected in human, orangutan, dog, and horse sequences, and the region was completely absent in opossum, chicken, and stickleback (Figure 1C).

To evaluate the homology of the *Cltc* microexon 33 sequence versus other species, we imported the sequence of the segment of the mouse *Cltc* gene between (but not including) exons 32 and 34 into the basic local alignment search tool (BLAST) available through the National Center for Biotechnology Information (NCBI). BLAST identified species that contained sequences with significant alignment to the mouse sequence input. Aligning the input mouse sequence for microexon 33 with the equivalent sequences in those identified species, revealed that (1) rodents exhibited the highest microexon 33 sequence homology (Figure 1D), especially between the mouse, rat, gerbil, vole, hamster, and deermouse species (Figure S3); (2) several bases differed between rodents and higher species (Figures 1C and 1D); (3) higher species, including humans, possessed an insertion within the microexon 33 sequence found in mice that could potentially generate an extended microexon 33 (containing 51 nt in humans) (Figures 1C and 1D, red bases between purple brackets); (4) some bases in the 5′ splice site of microexon 33 were not conserved (Figures 1C and 1D, red bases on the right); and (5) while most mice, rats, voles, and deermice had the consensus 3′ splice site (cag), the other identified species possessed a non-canonical (thus, weaker) 3′ splice site (caa instead of cag) (Figures 1C and 1D, red bases on the left). We suspect that the lack of conservation in the 3′ splice site is the main reason why microexon 33 is recognized and defined as an exonic region only in rats and mice but not in other species. Inspecting the intron downstream of exon 32 in the human *CLTC* gene revealed that 1,230 nt upstream of the 51-nt-long sequence that is highly homologous to the mouse *Cltc* microexon 33 (except the insertion), there is a 62-nt-long sequence flanked by a more canonical 3′ splice site (tag) that aligns poorly with the mouse *Cltc* sequence in that intronic area (Figure S4). Although the 62-nt-long sequence appears to be annotated in some human transcript assemblies, no information about the existence of the potential transcripts has been found in the databases.

### *Cltc* microexon 33 is species specific

We obtained RNA isolated from skeletal muscles of rats, mice, chickens, and humans during adulthood and neonatal or fetal stages and performed reverse transcription PCR (RT-PCR) assays using primers that targeted the constitutive exons 32 and 34 that flank microexon 33 (Figure 2A, red arrows denoting Cltc-38nt-f and Cltc-38nt-r primers), followed by separation of the products by polyacrylamide gel electrophoresis. Via densitometry, we quantified the level of microexon 33 inclusion, known as the percent spliced in (PSI) and defined as the percent of transcripts that include the exon over the total transcripts^60^^,^^61^ (Figure 2A, bottom). In chicken samples, we only detected one band (157 bp), which was expected and corresponded to the predicted transcripts lacking microexon 33 (Figure 2B, left). In humans, we also detected only one band (147 bp) corresponding to the predicted transcripts lacking the 51-nt-long sequence or the 62-nt-long sequence. We did not detect a band of 198 bp, which would have reflected the inclusion of the 51-nt-long sequence, nor did we detect a band of 209 bp, which would have reflected the inclusion of the 62-nt-long sequence (Figure 2B, right). In contrast, mouse and rat skeletal muscles showed the expected presence of two bands (+/− microexon 33) in different ratios depending on animal age. The PSI ranged from less than 10 in neonatal skeletal muscles to 69 or 48 in adult skeletal muscle of mice and rats, respectively (Figure 2C).

**Figure 2:**
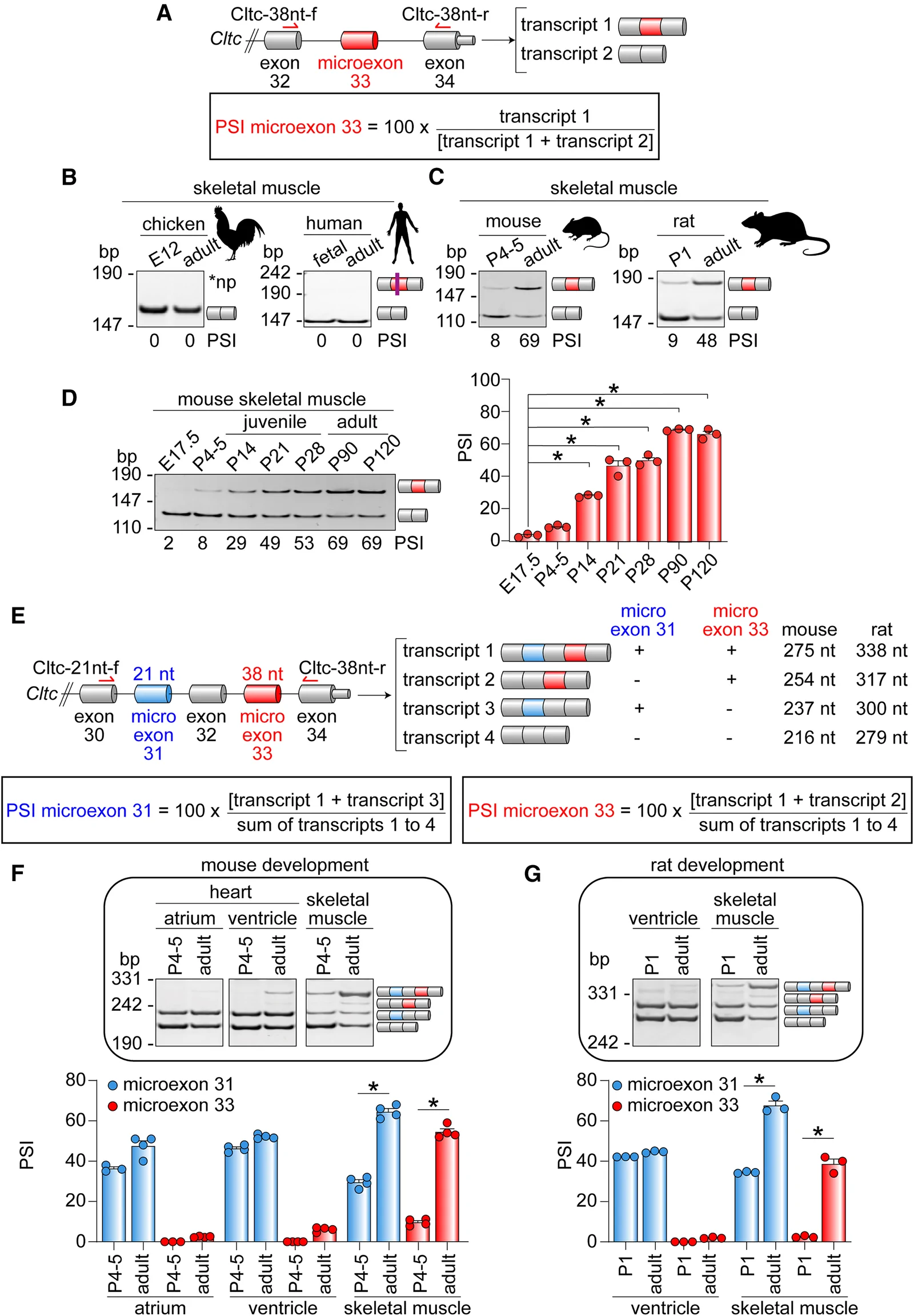
*Cltc* microexon 33 is species- and skeletal muscle specific (A) Schematic of the portion of the mouse *Cltc* gene between exons 32 and 34. Forward and reverse primers (Cltc-38-nt-f and Cltc-38-nt-r, respectively, red arrows) were designed to target the constitutive exons (gray) flanking the alternatively spliced microexon 33 (38 nt) (red). Formula used to calculate the level of microexon 33 inclusion (percent spliced in, PSI) (bottom). (B and C) Alternative splicing of *Cltc* microexon 33 was evaluated by reverse transcription PCR (RT-PCR) and polyacrylamide gel electrophoresis in skeletal muscles of chickens and humans (B) and mice and rats (C) at fetal or neonatal stages and in adulthood. In the cartoon of the longest predicted band in humans (+microexon 33), the vertical purple bar denotes the inserted sequence between the vertical purple bars presented in Figure 1D. Species silhouettes were obtained from PhyloPic open database under CC0 1.0 Universal Public Domain Dedication. (D) Alternative splicing of *Cltc* microexon 33 was assessed by RT-PCR and polyacrylamide gel electrophoresis in the mouse developing skeletal muscle between embryonic day 17.5 (E17.5) and postnatal day 120 (P120). The PSI was evaluated by densitometry. Results are shown as mean ± SEM. ∗*p* < 0.05, one-way ANOVA with Dunnett’s post hoc multiple comparison test, *n* = 3 biological pooled replicates for E17.5 (each replicate contained two hindlimb muscles pooled from two embryos), *n* = 3 biological pooled replicates for P4–5 (each replicate contained three gastrocnemius muscles pooled from three pups), and *n* = 3 biological replicates for P14, P21, P28, P90, and P120 (each replicate was obtained from one gastrocnemius muscle of one animal). bp, base pairs; np, not predicted (transcript and band are not predicted). (E) Schematic of the portion of the mouse *Cltc* gene between exons 30 and 34. Forward and reverse primers (Cltc-21-nt-f and Cltc-38-nt-r, respectively, red arrows) were designed to target the constitutive exons (gray) flanking both alternatively spliced microexons 31 (21 nt) (blue) and 33 (38 nt) (red). Expected PCR products are shown with their respective sizes for studies in mouse or rat tissues (right). Formulas used to calculate the PSI of microexon 31 or microexon 33 (bottom). (F) Alternative splicing of *Cltc* microexons 31 and 33 was evaluated by RT-PCR with Cltc-21-nt-f and Cltc-38-nt-r primers followed by polyacrylamide gel electrophoresis in neonate (P4–5; i.e., samples prepared from P4 and P5 animals were pooled together) and adult (4 months of age) mouse atrium, ventricle, and gastrocnemius muscle. The PSI of microexon 31 and the PSI of microexon 33 were evaluated by densitometry. Results are shown as mean ± SEM. ∗*p* < 0.05, two-way ANOVA with Tukey’s post hoc multiple comparison test, *n* = 3 biological pooled replicates for mouse P4–5 atrium (each replicate contained 18 atria pooled from nine pups), *n* = 4 biological pooled replicates for mouse adult atrium (each replicate contained two atria pooled from one mouse), *n* = 4 biological pooled replicates for mouse P4–5 ventricle and gastrocnemius (each replicate contained three gastrocnemius muscles or two ventricles pooled from three or two pups, respectively); *n* = 4 biological replicates for mouse adult ventricle and gastrocnemius. (G) Alternative splicing of *Cltc* microexons 31 and 33 was evaluated by RT-PCR followed by polyacrylamide gel electrophoresis in neonate (P1) and adult (3 months of age) rat ventricle and gastrocnemius muscle. The PSI of microexon 31 and the PSI of microexon 33 were evaluated by densitometry. Results are shown as mean ± SEM. ∗*p* < 0.05, two-way ANOVA with Tukey’s post hoc multiple comparison test, *n* = 3 biological replicates. bp, base pairs; nt, nucleotides. The splicing profiles of *Cltc* microexons 31 and 33 in mouse liver, intestine, spleen, kidney, lung, and brain tissues are shown in Figure S5A. The splicing profiles of *Cltc* microexons 31 and 33 in liver, gastrocnemius muscle, and cardiac ventricle in different mouse strains are shown in Figure S5B.

We next asked when microexon 33 starts to be included in the developing skeletal muscle. We used gastrocnemius muscle tissues collected from mice of different ages (E17.5, P4–5, P14, P21, P28, P90, and P120) to evaluate *Cltc* microexon 33 inclusion as described earlier. We observed that microexon 33 was mostly skipped in embryonic muscles and gradually included during postnatal development, reaching a plateau between P90 (mean PSI = 69) and P120 (mean PSI = 66) (Figure 2D).

In summary, our PCR results agree with the *in-silico* analysis presented in the previous sections and demonstrate that microexon 33 is only included in skeletal muscles of adult rats and mice and not in the other tested species. Moreover, microexon 33 alternative splicing is developmentally regulated in skeletal muscles in mice, being lowly included in fetal stages and reaching a plateau at around 3 months of age.

### *Cltc* microexon 33 inclusion is specific to skeletal muscle

We next asked whether *Cltc* microexon 33 is developmentally regulated in other murine tissues or, instead, whether this regulation is specific to skeletal muscle. Because we already knew that microexon 31 is developmentally regulated specifically in the heart and skeletal muscle (striated muscles) and mildly in the brain,^25^^,^^26^ we addressed our question using microexon 31 as a control. For the next set of RT-PCR assays, we designed primers targeting the constitutive exons 30 and 34 (Figure 2E, red arrows denoting Cltc-21nt-f and Cltc-38nt-r primers) to detect the four potential mouse *Cltc* transcripts: transcript 1 that contains 275 nt (includes both microexons), transcript 2 that contains 254 nt (−microexon 31, +microexon 33), transcript 3 that contains 237 nt (+microexon 31, −microexon 33), and transcript 4 that contains 216 nt (lacks both microexons) (Figure 2E, top). The PSI of microexon 31 was calculated as follows:

$$PSI\;microexon\;31=100\frac{transcript\;1\;abundance+transcript\;3\;abundance}{transcript\;1\;abundance+transcript\;2\;abundance+transcript\;3\;abundance+transcript\;4\;abundance}$$

The PSI of microexon 33 was calculated as follows:

$$PSI\;microexon\;33=100\frac{transcript\;1\;abundance+transcript\;2\;abundance}{transcript\;1\;abundance+transcript\;2\;abundance+transcript\;3\;abundance+transcript\;4\;abundance}$$

As shown in our previous studies,^25^^,^^26^ we observed that microexon 31 was regulated by alternative splicing to varying degrees in different mouse tissues: modestly in the heart (ventricles and atria), highly during skeletal muscle development, lowly in brain, and not regulated or regulated only to a nominal degree in liver, intestine, spleen, kidney, or lung (Figure 2F; Figure S5A, blue bars). In contrast, microexon 33 was highly regulated during development of skeletal muscle, very marginally in the heart, and not regulated in the other tested tissues (Figure 2F; Figure S5A, red bars). Furthermore, we confirmed that the tissue specificity of microexon 33 alternative splicing that we detected in FVB/N mice was recapitulated in two additional mouse strains commonly used in scientific research (129 S/SvEv and C57BL/6) (Figure S5B). Consistent with our findings in mice, we observed that microexon 33 was highly developmentally regulated in rat skeletal muscles and only marginally in rat ventricles (Figure 2G, red bars). Overall, these data suggest that mice and rats utilize tissue-specific regulation of *Cltc* alternative splicing to diversify the pool of *Cltc* transcript isoforms produced in skeletal muscles during development.

### Mouse Cltc transcripts that contain microexon 33 also include microexon 31

We next asked whether Cltc microexons 31 and 33, when included, were part of the same or different mouse transcripts. The answer to this question will inform our understanding of the ultimate consequences of alternative splicing in the potential encoded CLTC protein products. However, this is challenging to determine from the short-read RNA-sequencing data presented in Figure 1A. We therefore took two avenues to answer this question.

Our first approach used the results from the RT-PCR assays with primers targeting exons 30 and 34 because that design could detect the level of inclusion of each exon within the same transcript (Figure 2F). Analysis of the splicing patterns in skeletal muscles showed that the second band from the top was rarely detected (Figure 2F), indicating that transcripts including only microexon 33, without microexon 31, were nearly absent. Therefore, transcripts including microexon 33 almost always included microexon 31. Contrastingly, a considerable number of transcripts including microexon 31 did not include microexon 33 (Figure 2F, note the substantial abundance of the third band from the top).

To confirm these RT-PCR-derived conclusions, our second approach was to perform long-read RNA-sequencing in neonatal (P4–5) and adult (P256) mouse gastrocnemius muscles. Alignment of the long reads confirmed the presence of microexon 33, despite it being unannotated in the transcriptome, and demonstrated that this region was more included in the adult than in the neonatal skeletal muscle samples (Figure 3A, red box). Coverage analysis using deepTools^62^ revealed that the mean coverage depth (i.e., mean number of bases aligned over the region of interest) of *Cltc* microexon 33 was 18.0 in the neonatal samples and 28.0 in the adult samples (Figure 3B, bottom). This was striking because 315 reads mapped to the *Cltc* gene body in the neonatal samples, but only 117 reads did so in the adult samples (Figure 3B, top), indicating that despite the lower coverage, there is a persistent and detectable signal of microexon 33 inclusion in the adult skeletal muscles.

**Figure 3:**
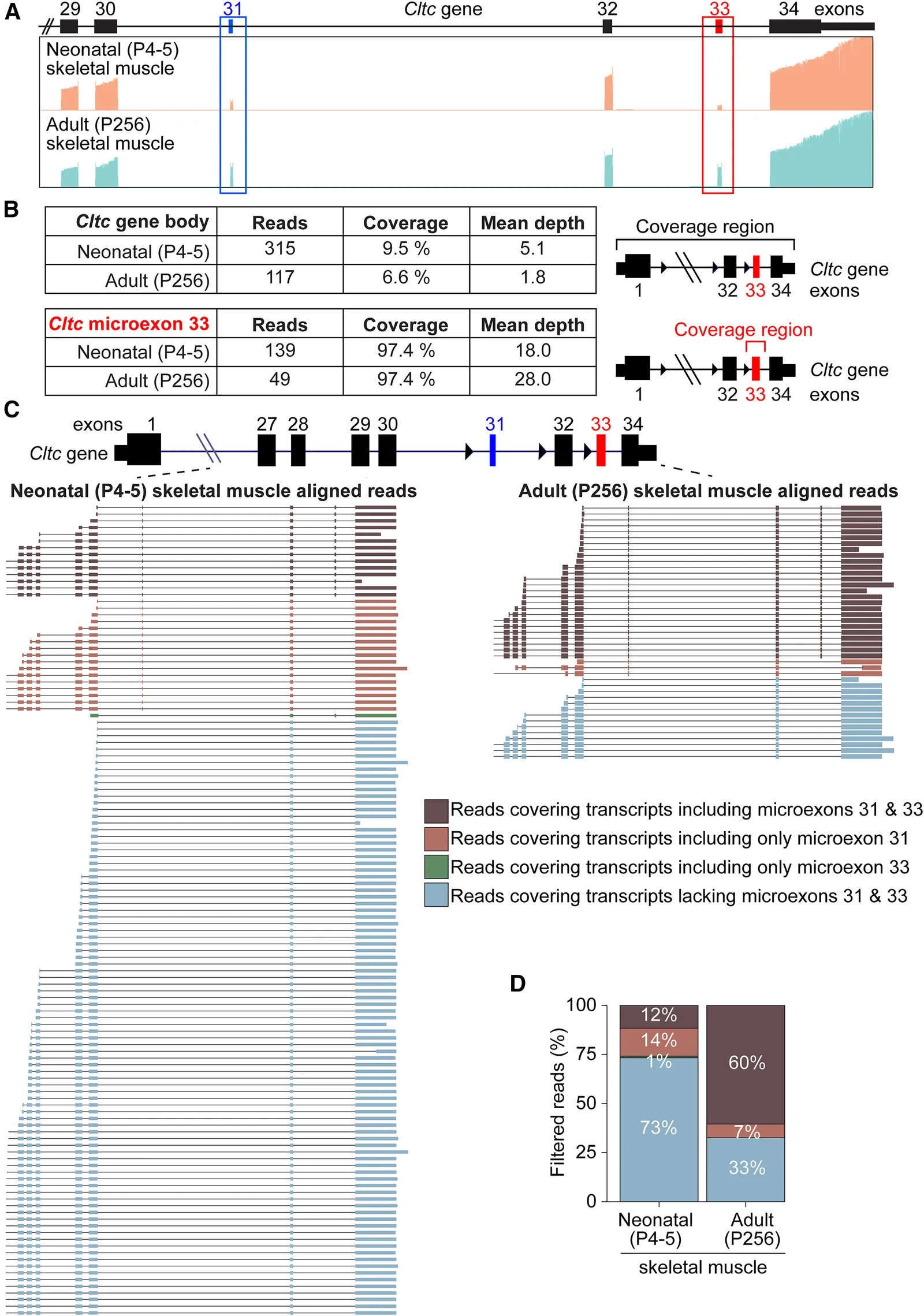
*Cltc* transcripts that contain microexon 33 also include microexon 31 (A) Neonatal (postnatal day 4 and 5, P4–5) and adult (P256) mouse gastrocnemius muscles were analyzed by long-read RNA-sequencing. Read coverage is shown across *Cltc* exons 29–34. (B) Read coverage statistics across the *Cltc* gene body (top) and *Cltc* microexon 33 (bottom). (C) Alignments of long reads mapping to *Cltc* in neonatal skeletal muscles (left) and adult skeletal muscles (right). (D) Distribution of filtered reads that mapped to each detected *Cltc* transcript isoform in neonatal and adult samples.

After filtering for *Cltc* reads spanning a minimum of the genomic interval containing microexons 31 and 33 to exclude reads that were truncated during sequencing, we visualized the long reads obtained from the neonatal and adult skeletal muscles (Figure 3C). Neonatal samples exhibited a clear preference for the Cltc isoform that skips both microexons (Figure 3C, left—light blue), whereas adult samples showed a strong preference for the Cltc isoform that includes both microexons (Figure 3C, right—dark brown). In agreement with the RT-PCR results, there was only one long read mapping to the Cltc isoform including microexon 33 but not microexon 31 (Figure 3C, left—green). We next quantified the number of reads that mapped to Cltc isoforms containing both microexons, only one of the microexons, or neither of them. We found that only 12% of the long reads mapped to the isoform that includes both microexons in the neonatal skeletal muscle samples, whereas 60% of the long reads mapped to the same isoform in the adult skeletal muscle samples (Figure 3D, dark brown), corroborating our RT-PCR results shown in Figure 2F.

In summary, the mouse *Cltc* microexon 33, when included, is nearly always part of transcripts that also contain microexon 31. This finding has implications for the potential encoded protein products (see next section).

### The mouse *Cltc* microexon 33 introduces a PTC that escapes NMD

Because microexon 31 contains 21 nt (multiple of three), its inclusion preserves the reading frame while encoding seven amino acids (VDAIKEK) that are inserted into the trimerization domain of the CLTC protein.^47^ *In-silico* studies have previously shown that the tripod quaternary structure is strengthened by microexon 31 inclusion due to an increase in coiled-coil interactions between the three α-helices of the trimerization domain.^47^ In contrast, microexon 33 contains 38 nt (not a multiple of three) and its inclusion introduces a PTC. PTCs have two major alternative outcomes. First, they can recruit the NMD machinery, leading to degradation of the PTC-containing transcripts. Second, if the transcript escapes NMD, translation can proceed by producing a protein isoform with a truncated C-terminus. Therefore, we hypothesized that the inclusion of *Cltc* microexon 33 would either trigger NMD of the transcripts in which this exon is included (no protein production) or produce a CLTC protein isoform with a shorter and distinct C-terminus. To discriminate between these two scenarios, we assessed the first possibility using the hSMG-1 inhibitor 11j (hSMG-1i) that inhibits the SMG-1 kinase, which is a critical component of the NMD pathway.^63^^,^^64^^,^^65^^,^^66^^,^^67^ We treated C2C12 myotubes with hSMG-1i or with dimethyl sulfoxide (DMSO, vehicle) for 24 h and then performed RT-PCR assays with the primers targeting *Cltc* exons 32 and 34. As a positive control, we used exon 4 (456 nt in length) of the serine/arginine-rich splicing factor 3 (*Srsf3*) gene because when included, exon 4 introduces a PTC in the transcripts that targets them to NMD^68^^,^^69^^,^^70^ (Figure 4A). As expected, treatment of myotubes with the NMD inhibitor for 24 h caused a dramatic increase in the abundance of Srsf3 transcripts that include exon 4 in comparison to DMSO-treated cells (Figure 4B, top band). Because C2C12 myotubes normally include *Cltc* microexon 33 at low levels (Figure 4C, first and third lanes), we reasoned that if transcripts containing this exon were subjected to NMD, treatment with hSMG-1i would lead to an increase in the intensity of the top PCR band (+microexon 33) as we observed for *Srsf3* exon 4. However, we did not detect an increase in the intensity of the top band (Figure 4C). We confirmed these qualitative differences between *Srsf3* exon 4 and *Cltc* microexon 33 by quantifying the abundance of each band (+/− exon) when cells were treated with the NMD inhibitor and normalizing it to the same band in the DMSO-treated cells. The abundance of Srsf3 transcripts including exon 4 was increased 17 times upon NMD inhibition (*p* = 0.03, hSMG-1i versus DMSO, *n* = 3) (Figure 4D, left), while the abundance of Cltc transcripts including microexon 33 did not increase, but rather slightly decreased (*p* = 0.003, hSMG-1i versus DMSO, *n* = 3) upon NMD inhibition (Figure 4D, right).

**Figure 4:**
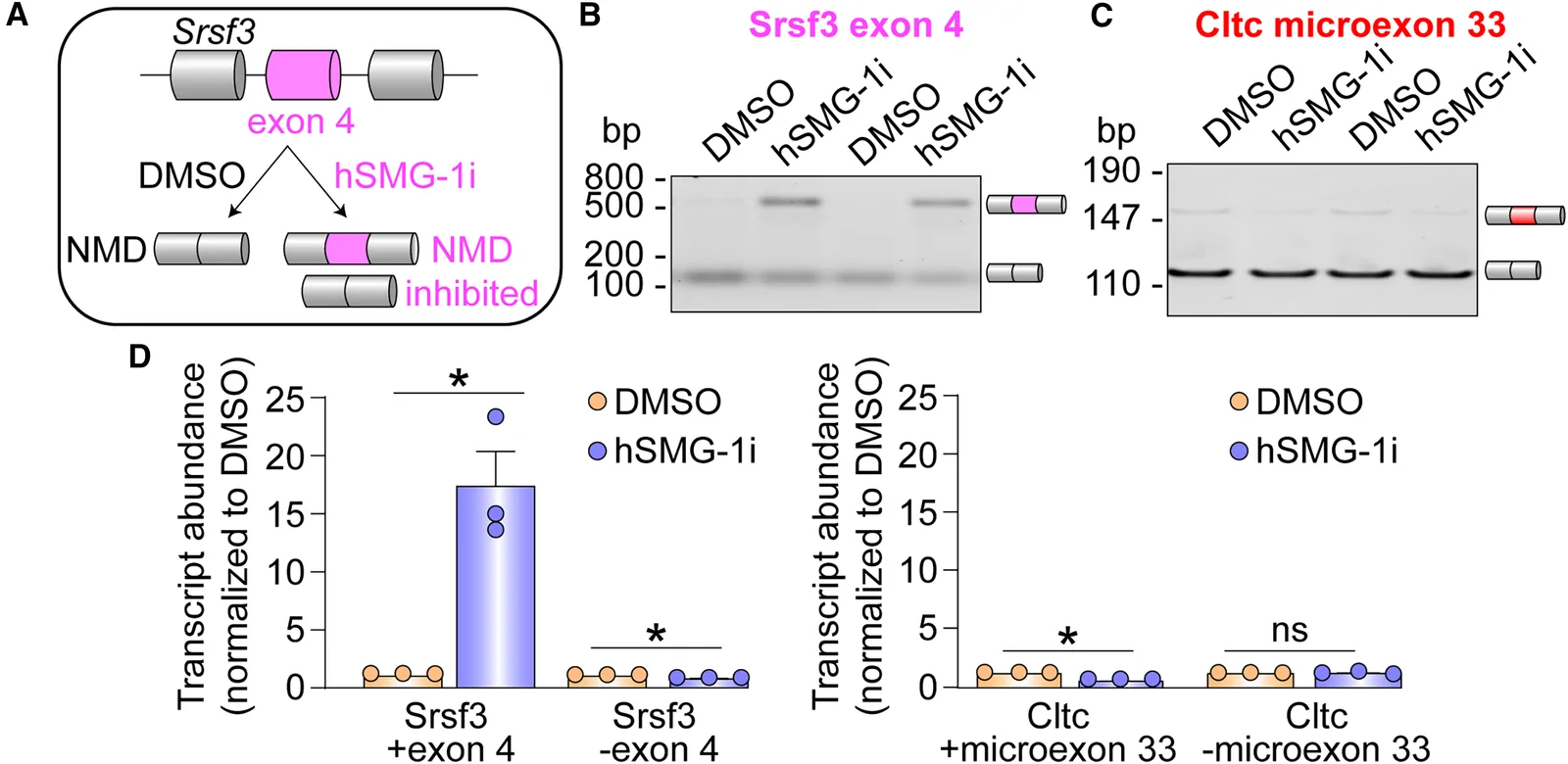
The mouse *Cltc* microexon 33 introduces a premature stop codon that escapes nonsense-mediated decay (A) Cartoon depicting alternative splicing of *Srsf3* exon 4 and its response to NMD inhibition. (B–D) C2C12 myotubes were treated with the NMD inhibitor hSMG-1i or DMSO (vehicle) for 24 h and the abundance of Srsf3 transcripts ± exon 4 (B) or Cltc transcripts ± microexon 33 (C) was evaluated by RT-PCR followed by agarose gel electrophoresis (B) or polyacrylamide gel electrophoresis (C). Abundance of each transcript (band) upon NMD inhibition was evaluated by densitometry and normalized to the abundance of the same transcript (band) upon DMSO treatment (D). Results are shown as mean ± SEM. ∗*p* < 0.05, unpaired Student’s *t* test, *n* = 3 biological replicates.

When PTCs are inserted <50 nt from the 3′ exon junction, transcripts tend to escape NMD.^71^^,^^72^ Because microexon 33 is the penultimate *Cltc* exon and is located within 50 nt of the 3′ exon junction, our results are in agreement with the literature. We conclude that transcripts containing microexon 33 might encode a CLTC protein isoform with a shorter C-terminus when compared to the canonical isoform.

### Inclusion of microexon 33 is predicted to generate a CLTC protein isoform with a truncated and distinct C-terminus

The canonical and most studied CLTC isoform lacks both microexons 31 and 33 and contains 1,675 amino acids (Figure 5A, second transcript from the top). Between 2014 and 2018, we reported the presence of *Cltc* microexon 31 in cardiac and skeletal muscles in mice and its regulation by alternative splicing in a tissue- and developmental-stage-specific manner.^4^^,^^5^^,^^25^ The inclusion of microexon 31 inserts seven amino acids (VDAIKEK) (Figure 5B, blue shaded region) after the lysine (K) residue located at position 1,609, producing a 1,682-amino-acid variant (Figures 5A and 5C, top isoform).

**Figure 5:**
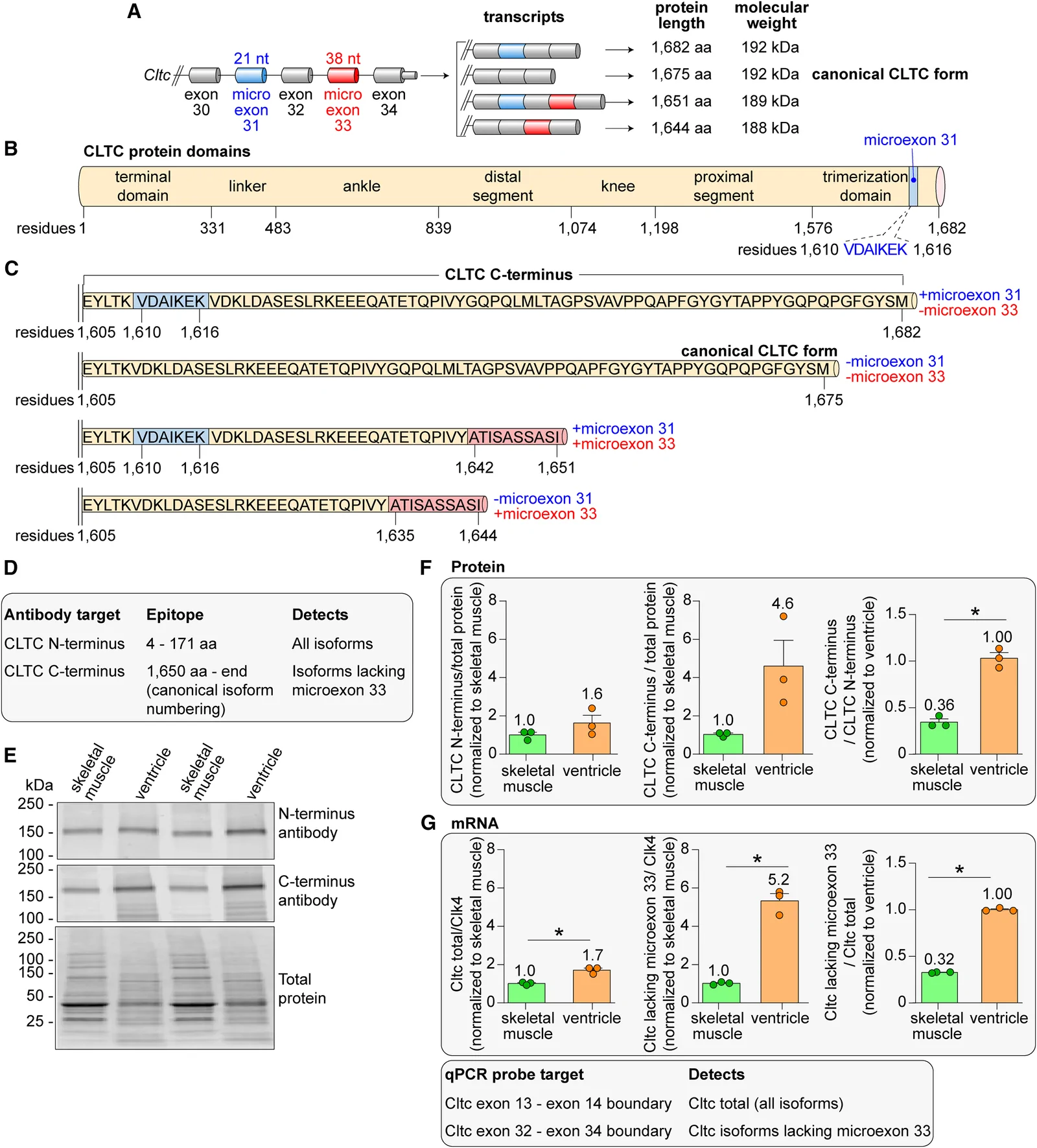
Inclusion of microexon 33 is predicted to generate a CLTC protein isoform with a short and distinct C-terminus (A) Schematic of the different Cltc transcripts and CLTC protein isoforms depending on inclusion or skipping of microexons 31 and 33. (B) CLTC domains based on literature. The peptide that is encoded by microexon 31 (blue) is inserted after the lysine (K) residue located at position 1,609. (C) Schematic of the CLTC C-termini for the four predicted protein splice variants in mouse. The first protein isoform (top row) includes the peptide produced by the inclusion of microexon 31 (VDAIKEK, blue), which is inserted after residue (lysine, K) 1,609. The second isoform (second row) that lacks both microexons 31 and 33 is the canonical CLTC form and contains 1,675 amino acids. Analysis of the microexon 33 sequence predicts that its inclusion causes: (1) a shift in the reading frame after residue (tyrosine, Y) 1,634 (numbering corresponds to the canonical form), (2) the translation of the peptide ATISASSASI (pink shaded region), and (3) the introduction of an earlier stop codon generating a shorter C-terminus (third row: isoform that includes both microexon 31 and microexon 33; fourth row: isoform that lacks microexon 31 but includes microexon 33). (D–F) An antibody targeting the CLTC N-terminus and an antibody targeting the CLTC C-terminus (D) were used to evaluate CLTC protein levels in ventricles and gastrocnemius muscles from adult (postnatal day 90, P90) mice via western blotting (E). Quantifications were performed by densitometry (F). (G) Cltc mRNA levels were measured in ventricles and gastrocnemius muscles of adult (P90) mice via quantitative real-time PCR (qPCR) using a probe targeting the exon 13-exon 14 boundary (left) (i.e., detecting total Cltc mRNA content) or a probe targeting the exon 32-exon 34 boundary (i.e., detecting only isoforms lacking microexon 33) (middle). CDC-like kinase 4 (*Clk4*) was used as housekeeping gene because its expression does not differ between the tested tissues. The left image shows the ratio between the abundance of Cltc mRNA lacking microexon 33 and the total abundance of Cltc mRNA. Results are shown as mean ± SEM. ∗*p* < 0.05, unpaired Welch’s *t* test, *n* = 3 biological replicates. CLTC protein downregulation during striated muscle development is shown in Figure S6, and CLTC arrangement in a striated pattern within skeletal muscle fiber cross-sections is shown in Figure S7.

Inclusion of the unannotated microexon 33 introduces a PTC that we predict might generate a CLTC protein isoform with a truncated and distinct C-terminus. Based on the nucleotide sequence, an ATISASSASI terminal peptide would begin after the tyrosine (Y) residue located at position 1,634 of the canonical CLTC isoform, generating a distinct, shorter C-terminus of the protein (Figure 5C, pink shaded region, bottom two isoforms).

The predicted molecular weights of the four possible CLTC protein isoforms range from 188 to 192 kDa (Figure 5A, right), making them indistinguishable by western blotting. No commercially available antibodies detect the novel, shorter CLTC C-terminus containing the ATISASSASI peptide. To circumvent these barriers, we took an indirect approach. We probed adult (P90) mouse cardiac ventricular and skeletal muscle tissues via western blotting using two distinct antibodies: (1) one targeting the CLTC N-terminus (epitope: residues 4–171) and (2) one targeting the long C-terminus (epitope: residues 1,650 to the end, numbering corresponding to the canonical isoform) downstream of where the truncation might occur (Figure 5D). We reasoned that the antibody targeting the N-terminus will recognize all CLTC isoforms, whereas the antibody targeting the C-terminus will recognize only those with the long C-terminus (+/− microexon 31, −microexon 33). Using the antibody targeting the N-terminus, we found that CLTC is 1.6 times more abundant in the adult ventricles than in the adult skeletal muscles of the same mice (Figure 5E, top blot; Figure 5F, left). This difference in CLTC protein abundance between these two adult tissues was close to the 1.7-fold difference detected by qPCR using a probe targeting the exon 13–exon 14 boundary (i.e., detecting all Cltc transcripts) (Figure 5G, left). In contrast, the immunoblots using the antibody targeting the C-terminus revealed that CLTC isoforms with the long C-terminus (+/− microexon 31, −microexon 33) were 4.6 times more abundant in the adult ventricles compared to the adult skeletal muscles of the same mice (Figure 5E, middle blot; Figure 5F, middle). Again, this finding was close to the 5.2-fold difference detected by qPCR using a probe targeting the exon 32–exon 34 boundary (i.e., detecting only transcripts lacking microexon 33) (Figure 5G, middle). Finally, we compared the ratio between the abundance of CLTC protein with the long C-terminus (detected by the antibody targeting the C-terminus) and the total CLTC abundance (detected by the antibody targeting the N-terminus) in both tissues. We found that in the adult skeletal muscles this ratio was ∼36% of that in the adult ventricles (Figure 5F, right). We observed nearly the same fraction at the mRNA level (Figure 5G, right).

Together, these results indicate that only a third of the CLTC protein present in adult mouse skeletal muscles contains the long C-terminus (−microexon 33) (detected by both antibodies) while approximately two-thirds contains the short, novel C-terminus (+microexon 33) (detected only by the N-terminal antibody). These proportions are in almost perfect agreement with, and can be explained by, our splicing results that showed that microexon 33 is included in ∼two-thirds of the transcripts (69% inclusion) in adult (P90) skeletal muscles (Figure 2D), while microexon 33 is very marginally included (∼2.4% inclusion) in adult ventricles (Figure 2F).

Knowing that *Cltc* microexon 33 is developmentally regulated, we next performed similar western blot assays with the N- and C-terminal antibodies comparing neonatal (P4–5) versus adult (P90) mouse skeletal muscles and ventricles. Using the N-terminal antibody, we found that CLTC levels in adult skeletal muscles are only 26% of those in neonates. Because microexon 33 PSI is ∼69% at P90, 31% of the CLTC protein content lacks this exon and can thus be detected by the C-terminal antibody. Therefore, we reasoned that from that 26% CLTC detected with the N-terminal antibody, the C-terminal antibody should only detect its 31% (26% × 31%/100 = 8%). Mathematically, this should be reflected by the C-terminal antibody showing that CLTC levels in adult skeletal muscles are ∼8% of those in neonates. Consistent with this prediction, our western blot assays revealed that adult skeletal muscles express only 7% of CLTC proteins that lack microexon 33 when compared with neonates (Figure S6A). In contrast, because microexon 33 inclusion is very low across development in the ventricles, we expected the N- and C-terminal antibodies to show very similar results to each other. Indeed, the experiments confirmed our prediction, showing that in adult ventricles, CLTC protein levels were only ∼22%–25% of those in neonates, independently of which antibody was used (Figure S6B).

In summary, this section shows indirect evidence that supports the production of a CLTC isoform with a shorter, distinct C-terminus as a consequence of the alternative inclusion of microexon 33 in a tissue- and developmental-stage-specific manner.

### CLTC is primarily arranged in a striated pattern within fiber cross sections

We were next curious whether the CLTC isoforms with different C-termini show distinct localization patterns in skeletal muscle tissue sections obtained from adult mice. Because there are no commercially available antibodies that detect the distinct ATISASSASI peptide, we used one antibody targeting the CLTC N-terminus (epitope: residues 869–919) and one antibody targeting the long C-terminus (epitope: residues 1,650 to the end, numbering corresponding to the canonical isoform) downstream of where the truncation might occur (Figure S7A). Staining with both antibodies showed a striated pattern that was observed in nearly all muscle fibers (Figure S7B, left). The distances between the striations, measured in identical fibers from serial subsequent cross sections, were remarkably similar between the two antibodies (Figure S7B, right). We thus concluded that CLTC is primarily arranged in a striated pattern within fiber cross sections, and that the presence or absence of microexon 33 does not seem to influence this localization pattern.

### Targeted mass spectrometry detects the expression of the shorter and distinct CLTC C-terminus

We next aimed to obtain direct evidence of the production of the CLTC isoform with the shorter and distinct C-terminus, containing the ATISASSASI peptide, via mass spectrometry. We used adult (P90) mouse skeletal muscle (gastrocnemius) and cardiac ventricle tissues to immunoprecipitate CLTC using an antibody that detects all isoforms (epitope resides within residues 200–300). We then analyzed the immunoprecipitated samples by liquid chromatography (LC)-mass spectrometry assays, where we targeted two tryptic peptides: the KEEEQATETQPIVYATISASSASI peptide (peptide 1) that should reflect the presence of CLTC isoforms including microexon 33, and the ALEHFTDLYDIK peptide (peptide 2) that should reflect the presence of all CLTC isoforms. The fragmentation patterns and retention times of the spectra in skeletal muscle and ventricle samples matched those obtained from synthetically produced peptide standards (Figure 6A). This result confirmed the proper detection of the CLTC protein portion that is common between all isoforms (by the surrogate peptide ALEHFTDLYDIK) and the production of a CLTC isoform with a shorter and distinct C-terminal sequence (KEEEQATETQPIVYATISASSASI) generated from the transcripts including microexon 33 in these tissues.

**Figure 6:**
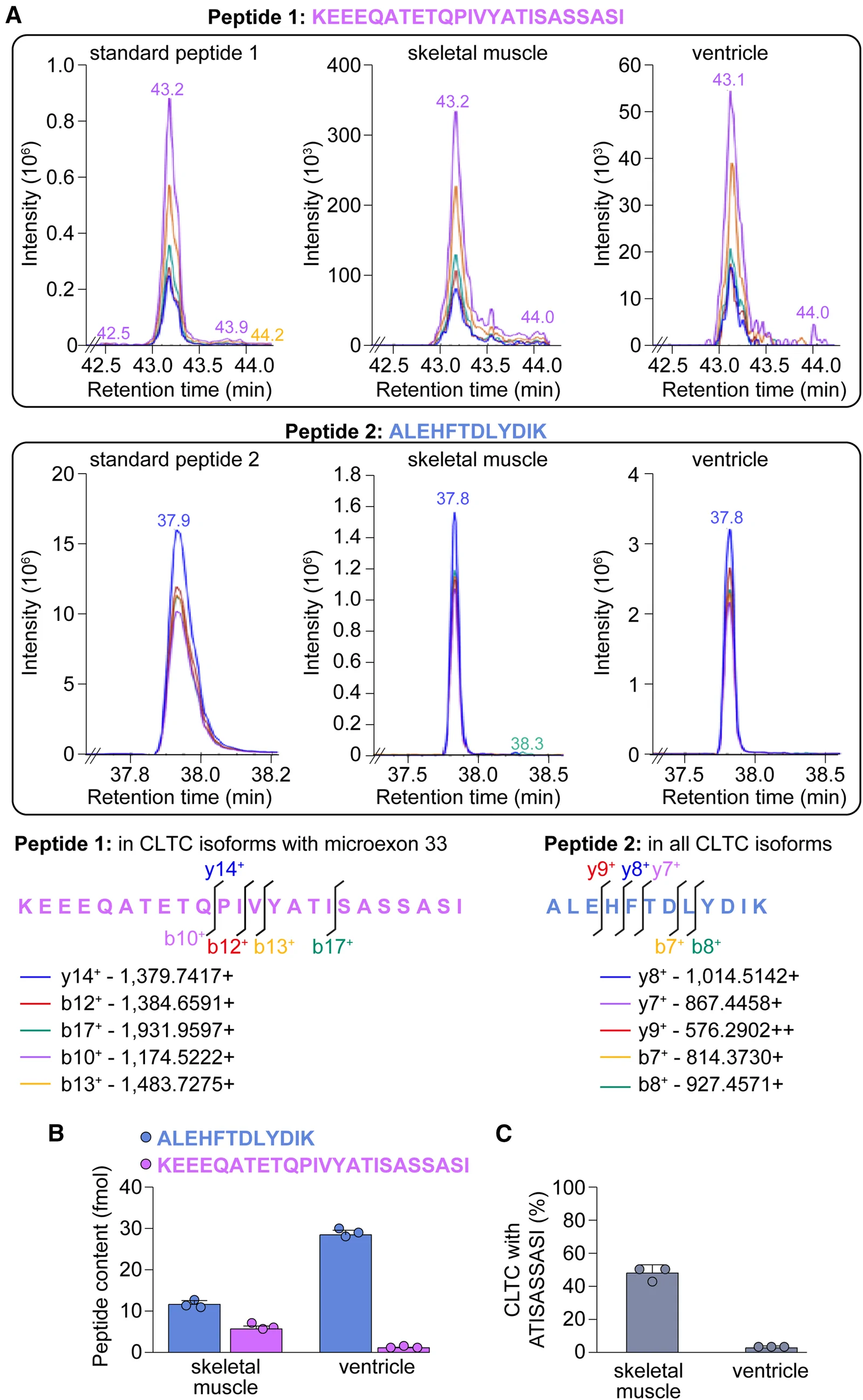
Targeted mass spectrometry detects the expression of the shorter and distinct CLTC C-terminus (A) Fragment ion chromatograms displaying the top five fragment ions of KEEEQATETQPIVYATISASSASI, precursor m/z 1,277.1320 (top), and ALEHFTDLYDIK, precursor m/z 732.8721 (bottom), obtained from the synthetic peptides 1 and 2 (left), mouse skeletal muscles (gastrocnemius) after immunoprecipitation of CLTC (middle), or mouse ventricles after immunoprecipitation of CLTC (right). (B) Content of the ALEHFTDLYDIK and KEEEQATETQPIVYATISASSASI peptides in mouse skeletal muscles (gastrocnemius) and ventricles. Results are shown as mean + SEM. *n* = 3 biological replicates. (C) Percentage of CLTC protein content with the ATISASSASI terminal sequence in mouse skeletal muscles (gastrocnemius) or ventricles calculated as 100 × (content of KEEEQATETQPIVYATISASSASI)/(content of ALEHFTDLYDIK). Results are shown as mean ± SEM. *n* = 3 biological replicates.

We next estimated the abundance of each peptide by comparing the area under the peaks of these spectra to serial dilutions of the synthetic peptides. Even when the ALEHFTDLYDIK peptide showed that immuno-enriched CLTC in the ventricle samples is 2.4 times more abundant than in the skeletal muscle samples, the abundance of CLTC isoforms including microexon 33 (measured by the KEEEQATETQPIVYATISASSASI peptide) was 6.4 times higher in the skeletal muscles than in the ventricles (Figure 6B). These abundance differences indicate that ∼47% of the CLTC protein immunoprecipitated from the mouse skeletal muscle samples contains microexon 33, whereas only ∼3% of the CLTC protein immunoprecipitated from the mouse ventricles contains the shorter C-terminus (Figure 6C). In conclusion, these data confirm both the identity of the shorter and distinct CLTC C- terminus and its robust expression in adult mouse skeletal muscle tissue.

### The mouse and rat skeletal muscle-specific CLTC isoform diverges from the canonical CLTC form at the same position that the human skeletal muscle-specific CLTCL1 protein does

The discovery of the unannotated *Cltc* microexon 33 led us to wonder why this penultimate exon is alternatively spliced exclusively in skeletal muscles of mice and rats. Humans and most vertebrates (except rodents) have, in addition to the *CLTC* gene, the *CLTCL1* gene, which is almost exclusively expressed in skeletal muscles (and at much lower levels in adipose tissue).^51^ In humans, CLTCL1 protein has been shown to play roles related to glucose metabolism that are critical for skeletal muscles and are not regulated by CLTC.^50^^,^^56^^,^^57^ Because glucose metabolism is highly conserved during evolution, the following question remains unanswered: which other genetic mechanisms might have evolved in rodents to diversify the menu of clathrin heavy chains and compensate for the loss of CLTCL1 protein? Driven by this question, we analyzed the sequence and organization of the mouse and human CLTC and CLTCL1 proteins and genes. The difference between the CLTCL1 and CLTC C-terminal regions begin at the same position (residue 1,634) where *Cltc* microexon 33 is inserted in the canonical CLTC isoform (Figure 7A). This divergence results in the final ATISASSASI peptide in the CLTC protein and the DFDGHE peptide in the CLTCL1 protein, both of which are positioned downstream of the residues in position 1,634 (tyrosine [Y] and phenylalanine [F], respectively) (Figure 7A). This was an intriguing finding: two genes, one in humans (*CLTCL1*) and the other in mice and rats (*Cltc*), are regulated by distinct mechanisms (gene duplication and alternative splicing, respectively) to ultimately produce similar diversification of the clathrin heavy chain protein C-termini.

**Figure 7:**
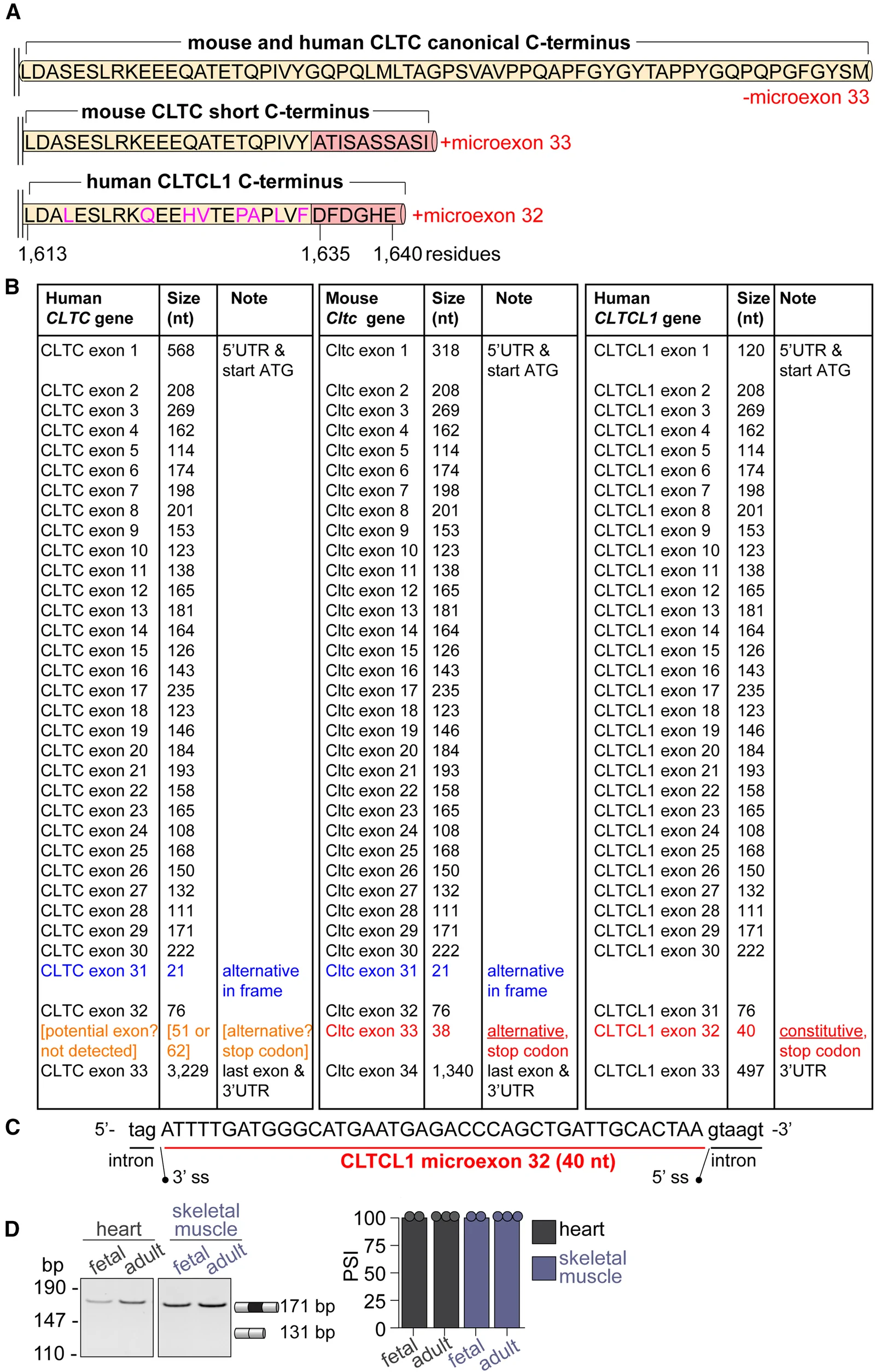
The mouse and rat skeletal muscle-specific CLTC isoform diverges from the canonical CLTC form at the same position that the human skeletal muscle-specific CLTCL1 protein does (A) Sequence alignment revealed that the human CLTCL1 C-terminus diverges from the canonical CLTC variant at the same position (residue 1,634) at which the peptide encoded by *Cltc* microexon 33 is inserted. Residues that are different between CLTCL1 and CLTC are shown in fuchsia. The peptide DFDGHE (pink shaded region) is encoded by the penultimate exon (40 nt) (microexon 32) of CLTCL1. (B) Exon-by-exon structure of the human *CLTC* gene (chart on the left), the mouse *Cltc* gene (chart on the middle), and the human *CLTCL1* gene (chart on the right). nt, nucleotides; UTR, untranslated region. (C) Sequence of the penultimate *CLTCL1* exon and its splice sites (ss). (D) Alternative splicing of *CLTCL1* microexon 32 (40 nt) was evaluated by reverse transcription PCR (RT-PCR) followed by polyacrylamide gel electrophoresis in commercially available RNA samples obtained from fetal and adult human skeletal muscle and heart. The level of exon inclusion (percent spliced in, PSI) was evaluated by densitometry. *n* = 3 biological replicates for adult human tissues; *n* = 2 for fetal human tissues.

The human *CLTCL1* gene, which contains 33 exons, the mouse *Cltc* gene, which contain 34 exons, and the human CLTC gene, which contains 33 exons, share several similarities and few differences. All three genes have exons with coding potential that contain the same number of nucleotides, and the sequences of the equivalent exons are very similar (Figure 7B); this explains the high (85%) homology between CLTC and CLTCL1 proteins. We noticed only two differences between the gene structures. First, the tissue- and developmental-stage-specific microexon 31 (21 nt) in the mouse *Cltc* and human *CLTC* genes is not present in the human *CLTCL1* gene (Figure 7B, blue exon). Second, the penultimate, alternative, skeletal muscle-specific microexon 33 (38 nt) in the mouse *Cltc* gene is not present in the human *CLTCL1* gene and is not strongly defined as an exonic region in the human *CLTC* gene. The human *CLTC* gene contains a 51-nt-long sequence very similar to the mouse *Cltc* microexon 33 (except the insertion) (Figure 1D) but flanked by a weak 3′ splice site and a 62-nt-long sequence different from the *Cltc* microexon 33 but flanked by a strong splice site. We did not detect the inclusion of either these sequences in human skeletal muscle transcripts (Figure 2B), therefore, these sequences appear not to be defined as exonic regions in the human *CLTC* gene. In the place of the mouse Cltc microexon 33, the human *CLTCL1* gene contains a 40-nt-long microexon (Figure 7B, right, red exon). Therefore, in both the mouse *Cltc* and human *CLTCL1* genes, the penultimate exons are out-of-frame microexons (38 nt and 40 nt) that introduce stop codons and thus generate shorter C-termini when compared to the canonical CLTC isoform (1,675 residue form). Analysis of the sequence of the penultimate *CLTCL1* microexon (40 nt) and its flanking intronic regions revealed the presence of canonical, strong splice sites: an ag in the 3′ splice site and a gta in the 5′ splice site (Figure 7C). This suggested that the *CLTCL1* 40-nt-long microexon is a constitutively spliced exon (i.e., is always included). We confirmed this by performing RT-PCR assays in human skeletal muscle and heart samples, where we observed only the band (171 bp) that corresponded to the transcripts that include the exon (Figure 7D). Therefore, the 40-nt-long microexon in CLTCL1 is always included (i.e., is constitutively spliced, not alternatively spliced) producing a 1,640-amino-acid-long CLTCL1 protein with a shorter C-terminus compared to the canonical 1,675-amino-acid-long CLTC isoform.

## Discussion

Despite their small size, microexons can have a large biological impact, particularly when they are included in a tissue-specific manner.^12^^,^^13^^,^^15^^,^^17^^,^^18^^,^^19^ However, most microexon research to date has focused on brain development and autism spectrum disorders.^12^^,^^14^^,^^17^^,^^19^^,^^22^^,^^23^^,^^24^ Very little is known about the existence, regulation, and function of microexons in other tissues. Here, we identified an unannotated microexon in the *Cltc* gene (microexon 33). This microexon differs from the previously reported *Cltc* microexon 31 in two main ways. First, whereas microexon 31 (21 nt) preserves the reading frame, does not introduce a stop codon, and generates a protein with a C-terminus that is identical to that of the canonical CLTC protein isoform, microexon 33 (38 nt) shifts the reading frame and introduces a PTC that escapes NMD and thus produces a truncated protein with a C-terminus that is distinct from that of the canonical CLTC isoform. Second, whereas microexon 31 inclusion has been reported to be specific to skeletal muscle, heart, and brain and is evolutionarily conserved among humans and rodents, microexon 33 inclusion is limited to skeletal muscles of adult mice and rats. In other tested species, including humans, CLTC transcripts in skeletal muscle do not include microexon 33 and we speculate that this can be due to the lack of strong splice sites. In mice and rats, the question that logically arises is: why is *Cltc* microexon 33 only included in skeletal muscles of mice and rats and not in other tissues and exclusively in adulthood? This splicing pattern suggests a regulatory mechanism where microexon 33 and/or its flanking intronic regions might be recognized by an RNA-binding protein (RBP) that is skeletal muscle specific (i.e., is highly expressed in skeletal muscle and is absent or lowly expressed in other tissues), that is upregulated in adulthood (versus earlier developmental stages), and that promotes exon inclusion. In such a model, the skeletal muscle-specific RBP would bind microexon 33 and/or its flanking intronic regions and promote exon inclusion exclusively when (adulthood) and where (skeletal muscle) that candidate RBP is expressed.

The human *CLTCL1* gene, which is highly homologous to the rodent *Cltc* gene, contains a penultimate exon (exon 32) that is a microexon similar in size (40 nt) to the mouse/rat *Cltc* microexon 33 (38 nt). However, unlike the rodent *Cltc* microexon 33, the human *CLTCL1* microexon 32 is constitutively spliced (i.e., is always included) due to the presence of consensus strong splice sites. Inclusion of *Cltc* microexon 33 in mice and rats or CLTCL1 microexon 32 in humans causes the translation of a CLTC or CLTCL1 protein, respectively, with C-termini that are shorter than that of the canonical CLTC variant. Therefore, while humans have diversified their pool of clathrin heavy chain protein isoforms by *CLTC*/*CLTCL1* gene duplication, mice and rats might have accomplished this via tissue-specific alternative splicing of the penultimate exon of the *Cltc* gene.

Brodsky and colleagues have previously revealed that the human *CLTC* and *CLTCL1* genes are located on paralogous regions of chromosomes 17 and 22, respectively, where several other adjacent paralogs also encode membrane trafficking proteins (for example, *MTMR4* and *MTMR3* genes, which encode myotubularin lipid phosphatases; or *AP2B1* and *AP1B1* genes, which encode subunits of the clathrin-associated adaptor proteins AP2 and AP1).^48^^,^^57^ Moreover, the clathrin light chains that bind CLTC to form the clathrin triskelion are also encoded by paralogous genes (*CLTA* and *CLTB*) on different chromosomes that arose from a local gene duplication mapped to the same time of the *CLTC/CLTCL1* gene duplication.^48^ *CLTCL1* was initially shown to be a pseudogene in mice^48^ and additional analyses did not detect this gene in 9 of 56 further jawed vertebrate genomes.^57^ In contrast, all vertebrate and non-vertebrate eukaryotes analyzed in that study retained the parent *CLTC* gene, establishing it as a gene under strong purifying selection,^57^ which is consistent with the ubiquitous functions of CLTC in clathrin-mediated endocytosis in all eukaryotes. In species where the *CLTCL1* gene is present, it is exclusively expressed in skeletal muscle (high levels) and adipose tissue (very low levels); in both tissues, CLTCL1 protein plays a role in the insulin-responsive GLUT4 trafficking.^50^^,^^56^^,^^57^ Some internal residues that are different between CLTCL1 and CLTC proteins were proposed to contribute to their distinct functions.^49^^,^^73^ One report highlights that CLTCL1 lacks the five QLMLT residues that are present in the long CLTC C-terminus where they serve as a recognition site for binding to the 70-kDa heat shock cognate protein (HSC70).^49^ *In vitro*, the HSC70-auxilin complex is less efficient in uncoating CLTCL1 assemblies versus CLTC assemblies, consistent with the possibility (although not demonstrated) that CLTCL1 does not efficiently bind HSC70 due to the lack of the QLMLT sequence.^49^ The authors thus proposed that CLTCL1 uncoating might be mediated by different proteins and/or mechanisms.^49^ However, the proposed proteins and mechanisms are still completely unknown as well as how the differences between CLTC and CLTCL1 C-termini molecularly contribute to their distinct functions.

Because CLTC is a vital protein with required functions in all organs, a clear understanding of the existing splice isoforms is significant in critical ways. While CLTC has been studied molecularly and cellularly for ∼45 years mostly in cell culture systems,^74^ skeletal muscle is one of the tissues in which CLTC plays not only canonical roles in endocytosis but also unconventional roles in regulating the intracellular architecture.^42^ The studies of CLTC splice isoforms and those of CLTCL1 protein have been confusing and challenging throughout the years in part due to two interrelated factors: (i) the lack of a clear understanding of the genes’ structure, the consequences of alternative splicing in the structure and abundance of the generated mature transcripts, and the variety and identity of the protein products; and (ii) confusion around results produced with antibodies. An important proportion of the antibodies available and, as such, used in published research target residues in the C-terminus of the canonical CLTC isoform that are located downstream where the divergence that we are reporting here occurs. As this divergence was not previously known to occur, the field has assumed that those antibodies recognized all CLTC proteins present in the samples. Therefore, when those antibodies were used in mouse or rat adult skeletal muscle, the proteins detected were the canonical but less predominant isoforms that contain the long C-terminus. This is important to consider because mice and rats are the most extensively used animal models in biomedical research. Antibodies targeting the N-terminus cannot distinguish between CLTC isoforms with different C-termini and there are no antibodies currently available to detect the distinct ATISASSASI peptide. Additionally, some antibodies thought to be specific to CLTCL1 or CLTC have been shown to cross-react between them and/or other unknown proteins leading to incorrect scientific conclusions. For example, CLTCL1 was reported to be expressed in rat skeletal muscle tissues based on immunofluorescence studies coupled to microscopy using an antibody targeting the trimerization domain of CLTCL1 (residues 1,521–1,640).^53^ However, this statement was proven to be incorrect when some of the same authors performed an evolutionary study of the *CLTCL1* gene learning that this is a pseudogene in rodents.^57^ One explanation for this missinterpreted finding is a nonspecific binding; but another one could be that the antibody that the authors used recognized the CLTC shorter C-terminus that we are reporting here and was not known at that moment. Nevertheless, this evidence highlights the importance of careful characterization of splice variants and their consequences in the production of diverse protein isoforms. Alternative splicing can be confusing, thus synergistic efforts between the experts on the proteins being regulated by this molecular mechanism and RNA researchers are certainly an avenue to pursue. Our study shields important light in this regard, specifically for the CLTC and CLTCL1 fields.

### Limitations of the study

The main limitation of our study is that it did not aim to determine the functional consequences of *Cltc* microexon 33 inclusion in adult rodent skeletal muscles. However, the discovery of this microexon and its regulation via alternative splicing in a species-, tissue-, and developmental-stage-specific manner will open multiple avenues of research for our group and other teams.

## Resource availability

### Lead contact

Requests for further information and resources should be directed to and will be fulfilled by the lead contact, Jimena Giudice (jimena_giudice@med.unc.edu).

### Materials availability

This study did not generate new unique reagents.

### Data and code availability

• RNA-sequencing dataset generated in this study has been deposited in the Gene Expression Omnibus (GEO) database with the accession number GEO: GSE333636. The proteomic dataset generated in this study has been deposited at the Proteomics Identification Database (PRIDE) repository with the accession number PRIDE: PXD082922. These deposited data are publicly available as of the date of this publication, and accession codes are listed in the key resources table.
• This article does not report original code.
• Any additional information related to the data reported in this article is available from the lead contact upon request.

## Acknowledgments

We thank the laboratories of Dr. Michael Bressan (chicken embryos), Dr. Kathleen Caron (non-FVB/N mouse strains), Dr. Sarah Cohen (rat pups), and Dr. Natasha Snider (adult rats) (all at The University of North Carolina at Chapel Hill) for their kind donation of animals used to collect tissues for this study. We thank Dr. Michael J. Emanuele (at The University of North Carolina at Chapel Hill) for the insightful discussion of protocols for CLTC immunoprecipitation. We acknowledge the technical support from the following core facilities at The University of North Carolina at Chapel Hill: High Throughput Sequencing Facility (10.13039/100031285HTSF) (supported by the University Cancer Research Fund, a Comprehensive Cancer Center Core Support Grant P30CA016086 from the National Institutes of Health (10.13039/100000002NIH), and the Center for Mental Health and Susceptibility Grant P30ES010126 from the NIH), the Histology Research Core Facility in the Department of Cell Biology and Physiology, the UNC Michael Hooker Metabolomics and Proteomics (MAP) Core (supported by the 10.13039/100008615UNC Lineberger Comprehensive Cancer Center Support Grant P30CA016086 from the 10.13039/100000002NIH and by the 10.13039/100011073UNC Nutrition Obesity Research Center Grant P30DK056350 from the NIH), the UNC Peptide Synthesis Core (RRID:SCR_017837) (supported by Grant P30CA016086 from the 10.13039/100000002NIH), and the Hooker Imaging Core, which is a member of the Carolina Microscopy (supported by the 10.13039/100008615UNC Lineberger Comprehensive Cancer Center Support Grant P30CA016086 from the NIH, the UNC College of Arts and Sciences, and the National Institute of Child Health and Human Development Intellectual and Developmental Disabilities Research Center Support Grant P50HD103573 from the NIH). This work was supported by the following funding sources: Ruth L. Kirschstein National Research Service Award Postdoctoral Individual Fellowships F32HL149147 (A.J.B.) and F32AR084326 (J.L.C.) and Ruth L. Kirschstein Institutional National Research Service Award T32CA244125 (A.K.), all from the 10.13039/100000002NIH; Graduate Research Fellowship DGE-2439854 from the 10.13039/100000001National Science Foundation (S.A.A.), Maximizing Investigators’ Research Award R35GM152426 from the 10.13039/100000002NIH (J.G.), CAREER Award #2239056 from the 10.13039/100000001National Science Foundation (J.G.), a Pilot & Feasibility Research Grant from the Nutrition and Obesity Research Center at The University of North Carolina at Chapel Hill (P30DK056350, 10.13039/100000002NIH) (J.G.), start-up funds from The University of North Carolina at Chapel Hill (J.G.), and funding provided by the Core Facilities Advocacy Committee and Office of Research Technologies at The University of North Carolina at Chapel Hill, School of Medicine (J.G.). The content is solely the responsibility of the authors and does not necessarily represent the official views of the 10.13039/100000002National Institutes of Health, the 10.13039/100000001National Science Foundation, or any other funding agency.

## Author contributions

Conceptualization, J.G.; methodology, R.E.B., A.J.B., J.L.C., S.A.A., A.K., S.P.L., J.A.E., S.C.B., D.D., and J.G.; investigation, R.E.B, A.J.B., J.L.C., S.A.A., A.K., S.P.L., J.A.E., S.C.B., D.D., and J.G.; validation, R.E.B, A.J.B., J.L.C., S.A.A., S.P.L., J.A.E., S.C.B., and J.G.; formal analysis, R.E.B, A.J.B., J.L.C., S.A.A., A.K., S.P.L., S.C.B., D.D., and J.G.; writing – original draft, R.E.B., J.L.C., and J.G.; writing – review and editing, R.E.B, A.J.B., J.L.C., S.A.A., A.K., S.P.L., J.A.E., S.C.B., D.D., and J.G.; visualization, R.E.B, A.J.B., J.L.C., S.A.A., A.K., S.C.B., D.D., and J.G.; funding acquisition, J.G.; resources, J.G.; supervision, S.C.B., D.D., and J.G.

## Declaration of interests

The authors declare no competing interests.

## STAR★Methods

### Key resources table

**Table:** 

REAGENT or RESOURCE	SOURCE	IDENTIFIER
**Antibodies**		

Mouse monoclonal IgG1 anti-clathrin heavy chain	BD Transduction Laboratories	Cat# 610499; RRID:AB_397865
Rabbit polyclonal IgG anti-clathrin heavy chain	Abcam	Cat# ab21679; RRID:AB_2083165
Rabbit polyclonal IgG anti-clathrin heavy chain	Invitrogen, Thermo Fisher Scientific	Cat# PA5-115024; RRID:AB_2899660
Rabbit recombinant monoclonal IgG clathrin heavy chain antibody [EPR12235(B)]	Abcam	Cat# ab172958; RRID:AB_2928108
Rabbit IgG isotype control recombinant antibody	Proteintech	Cat# 98136-1-RR; RRID:AB_3672282
Goat anti-rabbit IgG (H + L) secondary antibody, DyLight 800 4XPEG	Invitrogen, Thermo Fisher Scientific	Cat# SA5-35571 RRID:AB_2556775
Goat anti-mouse IgG (H + L) secondary antibody, DyLight 800 4XPEG	Invitrogen, Thermo Fisher Scientific	Cat# SA5-35521; RRID:AB_2556774
Goat anti-rabbit IgG (H + L) highly cross-adsorbed secondary antibody conjugated to Alexa Fluor Plus 647	Invitrogen, Thermo Fisher Scientific	Cat# A32733; RRID:AB_2633282

**Chemicals, peptides, and recombinant proteins**		

Dithiothreitol (DTT)	Sigma-Aldrich	Cat# D0632-10G
NuPAGE LDS buffer	Invitrogen	Cat# NP0007
2-Mercaptoethanol	Sigma-Aldrich	Cat# M6250
Nuclease free water	Invitrogen	Cat# AM9937
TRIzol reagent	Invitrogen	Cat# 15596026
DNase I	Qiagen	Cat# 79254
hSMG-1i	MedChem Express	Cat# HY-124719
Wheat germ agglutinin (WGA) conjugated to Alexa Fluor Plus 568	Invitrogen, Thermo Fisher Scientific	Cat# W56133
Antibody Diluent OP Quanto	Epredia	Cat# TA-125-ADQ
Fluoro-Gel II aqueous mounting medium containing DAPI	Electron Microscopy Services	Cat# 17985–50
10% neutral buffered formalin	Epredia	Cat# 5725
pH 6.0 retrieval buffer	Epredia	Cat# TA-135-HBL

**Critical commercial assays**		

High-capacity cDNA reverse transcription kit	Applied Biosystems	Cat# 4368813
TaqMan Fast Advanced Master Mix	Applied Biosystems	Cat# 4444556
Pierce BCA Protein Assay kit	Thermo Fisher Scientific	Cat# 23225
RNeasy fibrous tissue mini kit	QIAGEN	Cat# 74704
ZymoPURE Plasmid Miniprep kit	Zymo Research	Cat# D4210
Oxford Nanopore Direct RNA Sequencing Kit	Oxford Nanopore	Cat# SQK-RNA004
GoTaq Green Master Mix	Promega	Cat# M7123
TOPO TA cloning kit	Invitrogen	Cat# K452040
TaqMan Probe targeting Cltc exon 13 – exon 14 boundary; amplicon length: 111 nt	Thermo Fisher Scientific	Cat# Mm01303982_m1
TaqMan Probe targeting Cltc exon 32 – exon 34 boundary; amplicon length: 85 nt	Thermo Fisher Scientific	Cat# Mm01303974_m1
TaqMan Probe targeting Clk4 exon 1 – exon 2 boundary; amplicon length: 57 nt	Thermo Fisher Scientific	Cat# Mm00432585_m1

**Deposited data**		

Long RNA-sequencing data	This paper	GEO: GSE333636
Mass spectrometry data	This paper	PRIDE: PXD082922
RNA-sequencing data cardiomyocytes development	Giudice et al.^5^	GEO: GSE49906
RNA-sequencing data skeletal muscle development	Brinegar et al.^9^	GEO: GSE108402

**Experimental models: Cell lines**		

Mouse C2C12 cells	ATCC	Cat# CRL-1772, RRID:CVCL_0188

**Experimental models: Organisms/strains**		

Mouse: FVB/N	The Jackson Laboratory	Cat# 001800, RRID:IMSR_JAX:001800
Mouse: C57BL/6	The Jackson Laboratory	Cat# 000664, RRID:IMSR_JAX:000664
Mouse: 129 S/SvEv	The Jackson Laboratory	Cat# 012809, RRID: IMSR_JAX:012809
Rat: Sprague Dawley	Charles River	RRID:RGD_737891
Chicken: fertilized eggs	Pilgrim’s Farms, Sanford, NC, USA	N/A

**Oligonucleotides**		

*Mus musculus Cltc* alternative exon 38 nt primer forward: CGGAGTCGCTGAGAAAAGAAG	Sigma-Aldrich	N/A
*Mus musculus Cltc* alternative exon 38 nt primer reverse: AAAAGGTGCCTGGGGAGG	Sigma-Aldrich	N/A
*Rattus norvegicus Cltc* alternative exon 38 nt primer forward: CAGAGACACAGCCCATTGTTT	Sigma-Aldrich	N/A
*Rattus norvegicus Cltc* alternative exon 38 nt primer reverse: TCACATGCTGTACCCAAAGC	Sigma-Aldrich	N/A
*Gallus gallus Cltc* alternative exon primer forward: TGGATGCATCAGAGTCCTTGA	Sigma-Aldrich	N/A
*Gallus gallus Cltc* alternative exon primer reverse: CCGTATGGTGGTGCAGTGTA	Sigma-Aldrich	N/A
*Homo sapiens Cltc* alternative exon 51 nt primer forward: AGCTACAGAGACACAACCCA	Sigma-Aldrich	N/A
*Homo sapiens Cltc* alternative exon 51 nt primer reverse: CTGTACCCAAAGCCAGGC	Sigma-Aldrich	N/A
*Mus musculus Cltc* alternative exons 21 nt and 38 nt primer forward: GAAACCGCATGGAGACATAA	Sigma-Aldrich	N/A
*Mus musculus Cltc* alternative exons 21 nt and 38 nt primer reverse: AAAAGGTGCCTGGGGAGG	Sigma-Aldrich	N/A
*Rattus norvegicus Cltc* alternative exons 21 nt and 38 nt primer forward: GAAACTGCATGGAGACATAA	Sigma-Aldrich	N/A
*Rattus norvegicus Cltc* alternative exons 21 nt and 38 nt primer reverse: TCACATGCTGTACCCAAAGC	Sigma-Aldrich	N/A
*Mus musculus Srsf3* alternative exon 456 nt primer forward: TGGAACTGTCGAATGGTGAA	Sigma-Aldrich	N/A
*Mus musculus Srsf3* alternative exon 456 nt primer reverse: GAAAAGCTTCTCCTTCTTGG	Sigma-Aldrich	N/A
*Homo sapiens Cltcl1* alternative exon 40 nt primer forward: GCAAGAGGAGCATGTGACAG	Sigma-Aldrich	N/A
*Homo sapiens Cltcl1* alternative exon 40 nt primer reverse: ACGCGGAAGTTGTACATTGG	Sigma-Aldrich	N/A

**Software and algorithms**		

ImageJ version 1.51w for Windows	Schneider et al.^75^	https://imagej.net/RRID:SCR_003070
GraphPad Prism version 10.1.2	GraphPad Software	www.graphpad.com RRID:SCR_002798
QuantStudio^TM^ 7 Flex Real-Time PCR System	Applied Biosystems	Cat# 4485696
Image Studio^TM^ Software version 6.2.0.116	LI-COR Biotech	N/A
Image Lab version 6.0.1 for Windows	Bio-Rad	www.bio-rad.com RRID:SCR_014210
Deeptools v3.5.6	Ramirez et al.^62^	RRID:SCR_016366; https://deeptools.readthedocs.io/en/develop/
Samtools v1.23	Li et al.^76^	RRID:SCR_002105; https://samtools.sourceforge.net/
Bedtools v2.31.1	Quinlan et al.^77^	N/A
Minimap2 v2.30	Li et al.^78^	RRID:SCR_018550; https://github.com/lh3/minimap2
TimeTree	Kumar et al.^79^	RRID:SCR_021162; https://www.timetree.org
QuPath79 (version 0.5.1)	Bankhead et al.^80^	N/A
Fiji (version 2.16.0/1.54p)	Schindelin et al.^81^	RRID:SCR_002285; https://fiji.sc
Skyline (64-bit) 26.1	Pino et al.^82^	RRID:SCR_014080
PhyloPic	Open database under CC0 1.0 Universal Public Domain Dedication	RRID:SCR_019139; https://www.phylopic.org

**Other**		

Lysing Matrix D tubes	MP Biomedicals	Cat# 116930-CF
pUC19 ladder	Thermo Fisher Scientific	Cat# SM0221
GeneRuler 100-bp DNA ladder	Thermo Fisher Scientific	Cat# SM0243
Precision Plus Protein All Blue Standard	Bio-Rad	Cat# 4568084
Unstained Standard	Bio-Rad	Cat# 1610363
4–15% gradient Mini-Protean TGX Stain-Free gels	Bio-Rad	Cat# 4568084
0.45 μm Immobilon-FL PVDF membrane	Milipore Sigma	Cat# IPFL00010
Dynabeads Protein G magnetic beads	Invitrogen	Cat# 10007D
Charged DigiVU slides	Azer Scientific	Cat# 90DVAB-W+
C18 desalting columns	Thermo Fisher Scientific	Cat# 89852
Human fetal skeletal muscle RNA	Cell Applications	Cat# 1F60-50
Human fetal skeletal muscle RNA	Agilent Technologies	Cat# 540181
Human adult skeletal muscle RNA	Amsbio	Cat# R1234171-50
Human adult skeletal muscle RNA	Cell Applications	Cat# 1H60-50
Human adult skeletal muscle RNA	TakaRa	Cat# 636534
Human fetal heart RNA	Cell Applications	Cat# 1F30-50
Human fetal heart RNA	Agilent Technologies	Cat# 540165
Human adult heart RNA	Amsbio	Cat# R1234139-50
Human adult heart RNA	Cell Applications	Cat# 1H30-50
Human adult heart RNA	TakaRa	Cat# 636532

### Experimental model and study participant details

#### Cell lines and culture maintenance

C2C12 myoblasts (ATCC, CRL-1772) were cultured in Dulbecco’s Modified Eagle’s (DMEM) supplemented with 10% fetal bovine serum. When myoblasts reached 80–90% confluence, they were differentiated into myotubes for five days in DMEM supplemented with 2% horse serum.

#### Animal models

All animal studies were reviewed and approved for compliance with the regulatory guidelines of animal welfare by the Institutional Animal Care and Use Committee (IACUC) at The University of North Carolina at Chapel Hill (IACUC animal protocols: 24–239.0 (FVB/N mice), 24–049 (C57BL/6 and 129 S/SvEv mice), 23–118 (adult rats), and 23–202.0 (rat pups). All animals were housed in accredited facilities that were reviewed and inspected by the Association for Assessment and Accreditation of Laboratory Animal Care (AAALAC).

#### Mouse (*Mus musculus*) tissue collection

FVB/N mice were originally purchased from The Jackson Laboratory (JAX stock #001800) and then bred and maintained as a colony at The University of North Carolina at Chapel Hill. Adult C57BL/6 (P164) and 129 S/SvEv (P133) mice were donated for this study from colonies also maintained at The University of North Carolina at Chapel Hill. Time-mated pregnant dams were euthanized by isoflurane inhalant followed by cervical dislocation as a secondary euthanasia method on the 17^th^ day of gestation. E17.5 embryos were removed from the uterus and placed in ice-cold phosphate buffered saline (PBS). The placenta and amniotic sac were removed from the embryos, and both hind limbs were dissected after removing the foot from each limb. The skin surrounding the limb was removed and the skeletal muscle was snap frozen in liquid nitrogen. Neonatal (P4-5), juvenile (P14, P21, and P28), and adult (P90, P120, and P256) mice were euthanized by isoflurane inhalant followed by decapitation (P4-5) or cervical dislocation (P14, P21, P28, P90, P120, and P256) as a secondary physical method. The pelage surrounding the head and hind limbs was removed. The skull was removed to harvest the brain, and skeletal muscle was resected from the distal (gastrocnemius) hind limbs. The thoracic cavity was opened to harvest the heart and lungs, while the abdominal cavity was opened to collect liver, intestine, spleen, and kidneys. All tissues were blotted dry and snap frozen in liquid nitrogen.

#### Rat (*Rattus norvegicus*) tissue collection

Neonatal and adult skeletal muscles were donated for these studies from a Sprague Dawley colony maintained at The University of North Carolina at Chapel Hill. Neonatal (P1) and adult (3 months of age) rats were euthanized by carbon dioxide inhalation followed by decapitation (for neonates) or cervical dislocation (for adults). The thoracic cavity was opened to collect the heart. The pelage was removed from the hind limbs, and gastrocnemius muscles were harvested from both legs. All tissues were blotted dry before snap freezing in liquid nitrogen.

#### Chicken (*Gallus gallus*) RNA sources and tissue collection

Total RNA from adult chicken (7 months of age) skeletal muscle was purchased commercially (Zyagen, CR-102). Embryonic chicken skeletal muscle was donated for these studies from fertilized eggs (Pilgrim’s Farms, Sanford, NC, USA) incubated in a humidified chamber at 38°C until E12 based on previously established developmental staging.^83^ The eggs were windowed as previously described^84^ and skeletal muscle was excised from both legs and snap frozen in liquid nitrogen.

#### Human (*Homo sapiens*) RNA sources

Total RNAs from human fetal skeletal muscle (Cell Applications, #1F60-50; Agilent Technologies, #540181), human adult skeletal muscle (Amsbio, R1234171-50; Cell Applications, #1H60-50; TakaRa, #636534), human fetal heart (Cell Applications, #1F30-50; Agilent Technologies, #540165), human adult heart (Amsbio, R1234139-50; Cell Applications, #1H30-50; TakaRa, #636532) were purchased commercially.

### Method details

#### Genetic homology alignment and phylogenetic analysis

The intronic DNA sequence of the mouse *Cltc* gene between exons 32 and 34 was downloaded in FASTA format from the UCSC genome browser (mm39, transcript NM_001356393.1). This downloaded DNA sequence was then imported into BLAST (settings selected for Figure 1D: “Standard Database” - “Nucleotide collection”; settings selected for Figure S3: “Standard Database” - “RefSeq Genome Database”) maintained by the NCBI to identify sequences with significant alignment across species. Phylogenetic trees were obtained by TimeTree 5^79^ using the list of species from the BLAST alignment output. Species silhouettes were obtained from PhyloPic open database under CC0 1.0 Universal Public Domain Dedication.

#### RNA extraction from tissues and reverse transcription

Fresh frozen tissues were homogenized in lysing Matrix D tubes (MP Biomedicals, #116930-CF) containing 1.4 mm zirconium-silicate beads and and TRIzol reagent (Invitrogen, #15596026). Complete lysis was achieved by applying the following parameters in the Precellys 24 homogenizer (Bertin Instruments): (1) 6,500 rpm for 25 s; (2) 15 s pause; and (3) 6,500 rpm for 25 s. RNA was extracted from the tissue lysates with TRIzol reagent following the manufacturer’s recommended protocol. RNA concentrations were measured in a NanoDrop Lite spectrophotometer (Thermo Fisher Scientific). RNA templates (2 μg) were reverse transcribed using the High-capacity cDNA reverse transcription kit (Applied Biosystems, #4368813) according to the manufacturer’s recommended protocol. Thermocycler conditions for cDNA synthesis were as follows: (1) 25°C for 10 min; (2) 37°C for 2 h; (3) 85°C for 5 min; and (4) 4°C pause.

#### Molecular cloning and sequencing

PCR amplified cDNA from an adult (P120) FVB/N mouse gastrocnemius muscle was cloned into the pCR 4-TOPO vector and transformed into competent *E. coli* DH5α cells using the TOPO TA cloning kit (Invitrogen, K452040) according to the manufacturer’s recommended protocol. Transformed cells were cultured on Luria-Bertani (LB) agar plates containing 100 μg/mL ampicillin overnight at 37°C. The next day, six single colonies were individually selected and cultured overnight in LB broth supplemented with 100 μg/mL ampicillin at 37°C. Plasmid DNA was extracted from grown cultures and purified using a ZymoPURE Plasmid Miniprep kit (Zymo Research, D4210) following the manufacturer’s instructions. Purified DNA was quantified using a NanoDrop Lite spectrophotometer (Thermo Fisher Scientific). A DNA aliquot (500 ng) was sequenced by the Sanger method (Genewiz) using the universal M13 forward and M13 reverse sequencing primers (M13F and M13R, respectively).

#### Alternative splicing analysis by PCR

The Primer3 (version 4.1.0) web design tool^85^ was used to design primers (Table S1) targeting the constitutive regions flanking the alternatively spliced exons of interest. Primers were diluted to a final 0.5 μM concentration in GoTaq Green Master Mix (Promega, M7123) and nuclease-free water (Invitrogen, AM9937). PCR reactions were performed as follows: (1) 95°C for 75 s; (2) 27 cycles of 95°C for 45 s, 57°C for 45 s, 72°C for 1 min; (3) 72°C for 10 min; and (4) 25°C pause. PCR products were resolved via 6% polyacrylamide gel electrophoresis using TAE buffer (40 mM Tris, 20 mM acetic acid, 1 mM EDTA pH 8.5) for 3–4 h at 150–160 V. Polyacrylamide gels were stained with 0.4 μg/mL ethidium bromide for 10 min. All gels were imaged under ultraviolet illumination in a ChemiDoc XRS+ Imaging System (Bio-Rad). Alternative splicing was quantified by densitometry using the Image Lab software (version 6.0.1, Bio-Rad). The product length-dependent staining bias of ethidium bromide was corrected using a standard curve based upon the pUC19 ladder (Thermo Fisher Scientific, SM0221). The PSI was calculated as previously described.^2^

#### Long-read RNA-sequencing

Gastrocnemius muscles were dissected from neonatal (P4-5) and adult (P256) FVB/N mice. Tissues were homogenized as described above in RLT buffer supplemented with 0.143 M 2-mercaptoethanol (Sigma-Aldrich, M6250). RNA was extracted using the RNeasy Fibrous Tissue Mini kit (Qiagen, #74704) and treated with DNase I (Qiagen, #79254) following the manufacturer’s recommended protocol. RNA integrity was assessed on a TapeStation platform and only RNA samples with RNA Integrity Number (RIN) > 9 were advanced to library preparation. Where required by the kit, inputs were prepared as 1 μg total RNA per library. Libraries were prepared using the Oxford Nanopore Direct RNA Sequencing kit (SQK-RNA004) following the manufacturer’s protocol. Briefly, sequencing adapters and motor protein were ligated to the RNA, and a short reverse-transcription step was performed to generate an RNA–cDNA hybrid to improve sequencing stability. Libraries were purified according to the protocol and quantified prior to loading. Each library was loaded onto a MinION Flow Cell (RNA) (FLO-MIN004RA), and sequencing was performed on a GridION using MinKNOW with settings recommended for RNA004 direct RNA runs. Run duration and termination criteria (e.g., time or active pore threshold) followed standard laboratory practice and ONT guidance. Raw signal data were collected in MinKNOW and basecalled in MinKNOW and later offline with Dorado using an RNA004 SUP model (e.g., rna004_130bps_sup@v5.x) consistent with the current ONT RNA004 model family.

#### Analysis of long-read RNA-sequencing data

Raw ONT dRNA fastq files were merged into one file per sample. Reads were aligned to the mm9 mouse genome assembly using minimap2 v2.30.^78^ The parameters were set to “-ax splice -uf -k14 -B3 -O3,20” based on the recommended options for ONT dRNA-seq and mismatch penalties for aligning to microexons. Alignments were sorted and indexed using samtools v.1.23.^76^ The bedGraph files (used for visualization) were generated with the bamCoverage function from the deeptools v3.5.6 software^62^ with binSize 1 and region chr11:86508154:86570994. Read coverage was visualized using the UCSC Genome browser.^86^ Samtools v1.23^76^ and bedtools v2.31.1^77^ were used to generate bed files to visualize and quantify exon alignments across reads. For analysis and isoform mapping statistics, long reads were filtered to include those with start sites and end sites spanning a minimum genomic interval including microexons 31 and 33 to exclude prematurely truncated reads. Figure panels related to long-read RNA-sequencing were assembled using R v4.5.0 and Adobe Illustrator.

#### NMD studies

C2C12 myoblasts were cultured in DMEM supplemented with 10% fetal bovine serum. When myoblasts reached 80-90% confluence, they were differentiated into myotubes for five days in DMEM supplemented with 2% horse serum. At differentiation day 5, myotubes were treated with 0.5 μM hSMG-1i (MedChem Express, HY-124719) or the vehicle DMSO for 24 h. RNA was then isolated using TRIzol reagent (Invitrogen, #15596018) as detailed above for the tissue samples. RT-PCRs were performed as described above for the tissues, but PCR products for Srsf3 exon 4 were resolved via 1.5% agarose gel (0.5 μg/mL ethidium bromide) electrophoresis using TAE buffer for 1.5 h at 110 V and the GeneRuler 100-bp DNA ladder (Thermo Fisher Scientific, SM0243). Abundance of Cltc transcripts ± microexon 33 and Srsf3 transcripts ± exon 4 was quantified by densitometry using the Image Lab software (version 6.0.1, Bio-Rad).

#### Preparation of protein lysates from murine tissues

Ventricles and gastrocnemius muscles isolated from male adult (P90) mice were weighed and placed in Lysing matrix D tubes (MP Biomedicals, #116930-CF) with RIPA buffer (50 mM Tris-HCl pH 8.0, 150 mM NaCl, 5 mM EDTA pH 8.0, 0.1% sodium dodecyl sulfate (SDS), 0.5% sodium deoxycholate, 1% NP-40) supplemented with protease and phosphatase inhibitors (Millipore Sigma). Tissues were homogenized using a Precellys-24 homogenizer (Bertin Instruments) with the following settings: (i) 6,500 rpm for 25 s; (ii) 15 s pause; and (iii) 6,500 rpm for 25 s. Lysates were centrifuged at 10,000 rpm for 3 min (4°C) and transferred into new tubes. Lysates were sonicated in an ice bath for 3 min (30 s on, 30 s off) at 75 V and then centrifuged at 18,000 × g for 10 min (4°C). Protein concentration was estimated using the Pierce BCA Protein Assay kit (Thermo Fisher Scientific, #23225).

#### Western blotting

Protein samples (20–40 μg) were boiled at 95°C for 5 min in loading buffer (50 mM Tris-HCl pH 6.8, 12.5 mM EDTA pH 8.0, 10% glycerol, 2% SDS, 0.02% bromophenol blue, 360 mM beta-mercaptoethanol). Denatured proteins were loaded into 4–15% gradient Mini-Protean TGX Stain-Free gels (Bio-Rad, #4568084) together with the Precision Plus Protein All Blue (Bio-Rad, #1610373) and Unstained (Bio-Rad, #1610363) standards. Proteins were resolved by electrophoresis at 90 V for 90 min in 25 mM Tris, 192 mM glycine, and 3.5 mM SDS. Proteins were transferred (100 V for 1 h) onto a 0.45 μm Immobilon-FL PVDF membrane (Millipore Sigma, IPFL00010) using a buffer containing 25 mM Tris, 192 mM glycine, and 20% methanol. After transfer, total proteins were visualized by UV light using the ChemiDoc XRS+ Imaging System (Bio-Rad). Membranes were blocked for 1 h (room temperature) with 5% non-fat dried milk in 19 mM Tris pH 7.6, 2.7 mM KCl, 137 mM NaCl, 0.1% Tween 20 (TBST). Membranes were then incubated at 4°C overnight with the following primary antibodies diluted 1:1,000 in TBST containing 5% bovine serum albumin (BSA): (i) mouse monoclonal IgG1 anti-clathrin heavy chain (epitope: residues 4–171) (BD Transduction Laboratories, #610499), or (ii) rabbit polyclonal IgG anti-clathrin heavy chain (epitope: residues 1,650 to the end – numbering from the canonical CLTC isoform) (Abcam, ab21679). The next day, membranes were washed three times with TBST (10 min each) and then incubated for 90 min at room temperature with either a goat anti-rabbit IgG (H + L) secondary antibody, DyLight 800 4XPEG (Invitrogen, SA5-35571) or a goat anti-mouse IgG (H + L) secondary antibody, DyLight 800 4XPEG (Invitrogen, SA5-35521) diluted 1:10,000 in a 5% non-fat-dried milk in TBST solution. Membranes were washed with TBST three times (10 min each) and then imaged on the Odyssey CLx Blot Imager (Li-Cor). The intensity of the detected CLTC bands was quantified by densitometry using Image Studio (Li-Cor) software and normalized to the transferred total protein (quantified by ImageJ software^75^ or the Image Lab version 6.0.1 from Bio-Rad).

#### qPCR

RNA extracted from adult (P90) mouse gastrocnemius muscle or ventricle was converted into cDNA using the High-capacity cDNA Reverse Transcription kit (Applied Biosystems, #4368813) as described above. qPCRs were then performed using the cDNA (50 ng) and the TaqMan Fast Advanced Master Mix (Applied Biosystems, #4444556) with the following TaqMan probes (Thermo Fisher Scientific): (i) Mm01303982_m1 (targeting Cltc exon 13 – exon 14 boundary; amplicon length: 111 nt), (ii) Mm01303974_m1 (targeting Cltc exon 32 – exon 34 boundary; amplicon length: 85 nt), and (iii) Mm00432585_m1 (targeting Clk4 exon 1 – exon 2 boundary; amplicon length: 57 nt). qPCR reactions were performed in quadruplicate in MicroAmp fast 96-well plates (Applied Biosystems, #4346907) and the following thermocycler settings: 40 cycles of (1) 50°C for 2 min; (2) 95°C for 20 s; (3) 95°C for 1 s; and (4) 60°C for 20 s. Results were quantified using a QuantStudio 7 Flex Real-Time PCR System (Applied Biosystems). Clk4 was used as housekeeping gene to determine the relative mRNA abundance, and the fold changes were calculated by applying the delta-delta cycle threshold (ΔΔC_T_) quantification method.

#### Immunofluorescence and microscopy of skeletal muscle tissue sections

Gastrocnemius muscles were dissected from male C57BL/6 mice (58–61 weeks of age), pinned to corkboard at their natural lengths (not shortened or stretched), and fixed in 10% neutral buffered formalin (Epredia, #5725) for ∼25 h at room temperature. Tissues were then washed twice with PBS (pH 7.4) and submerged in 70% ethanol until processing. Samples were processed on the standard cycle at 60°C in a Sakura Tissue-Tek VIP 5 Tissue Processor for ∼8.5 h. After processing, samples were embedded using the Leica EG1160 Embedding Station. Tissue blocks were serially sectioned using a Leica RM2235 Microtome, and sections (5 μm) were floated on a water bath before being placed on charged DigiVU slides (Azer Scientific, #90DVAB-W+). Slides were air dried overnight at room temperature, and baked at 60°C for 1.5 h and then at 50°C overnight. After resting at room temperature for 24 h, slides were deparaffinized and hydrated using xylene (2 × 5 min), absolute ethanol (5 min), 95% ethanol (4 min), 70% ethanol (3 min) and distilled water (2 × 2 min). Heat induced epitope retrieval (HIER) was performed using pH 6.0 retrieval buffer (Epredia, TA-135-HBL), followed by cooling for 20 min and rinsing with deionized water twice (2 min each). After rinsing for 2 min in Tris-buffer (pH 7.6), tissues were blocked for 1 h in 10% goat serum, and then incubated with rabbit polyclonal IgG anti-clathrin heavy chain diluted 1:200 (epitope: residues 869–919) (Invitrogen, PA5-115024) or rabbit polyclonal IgG anti-clathrin heavy chain diluted 1:50 (epitope: residues 1,650 to the end – numbering from the canonical CLTC isoform) (Abcam, ab21679) overnight at 4°C in a humidified chamber. Tissues were rinsed twice (2 min each) with Tris-buffer (pH 7.6) containing 0.02% Tween and once (2 min) with Tris-buffer (pH 7.6). Then, tissues were incubated in goat anti-rabbit IgG (H + L) highly cross-adsorbed secondary antibody conjugated to Alexa Fluor Plus 647 diluted 1:1,000 (Invitrogen, A32733) for 2 h at room temperature. Slides were rinsed twice (2 min each) with Tris-buffer (pH 7.6) containing 0.02% Tween and then with Tris-buffer (pH 7.6) (2 min). The plasma membrane of the muscle fibers was stained with wheat germ agglutinin (WGA) conjugated to Alexa Fluor Plus 568 diluted 1:1000 (Invitrogen, W56133) for 1 h at room temperature. Primary and secondary antibodies as well as WGA were all diluted in Antibody Diluent OP Quanto (Epredia, TA-125-ADQ). Finally, slides were rinsed twice (2 min each) with Tris-buffer (pH 7.6) containing 0.02% Tween followed by another rinse (2 min) with Tris-buffer (pH 7.6), cover-slipped using Fluoro-Gel II aqueous mounting medium containing DAPI (Electron Microscopy Services, #17985–50), and imaged on a VS200 slide scanner (Olympus/Evident) with a 40×/0.95NA objective and the following excitation settings and epifluorescence filters: DAPI (10% power; 4.69 ms exposure; excitation center wavelength 378 nm; emission center wavelength 447 nm), TRITC/Cy3 (WGA staining) (50% power; 80.05 ms exposure; excitation center wavelength 545 nm; emission center wavelength 609 nm), and Cy5 (CLTC staining) (100% power; 548.31 ms; excitation center wavelength 635 nm; emission center wavelength 680 nm). Images were viewed and analyzed using QuPath79 (version 0.5.1)^80^ and Fiji (version 2.16.0/1.54p).^81^

To quantify the distance between the striations detected by each clathrin heavy chain antibody, identical fields of view containing ∼20 muscle fibers were identified in serial, subsequent sections and then cropped. The three channels were then separated and only the Cy5 channel (shown in red, detecting the clathrin heavy chain) was processed using the follow pipeline in Fiji: (i) the background was subtracted (rolling ball method, radius: 10 pixels); (ii) a median filter was applied (radius: 2 pixels); local contrast was enhanced by the Contrast Limited Adaptive Histogram Equalization (CLAHE) method (block size: 49, histograms bins: 256, maximum slope: 3.00, no mask); and a second median filter was applied (radius: 4 pixels). The three channels were then merged as a composite image. With the Cy5 channel selected, a line (width: 20) was drawn crossing and perpendicularly to at least three striations and fully contained within one fiber. A fluorescence intensity profile of the line was plotted and analyzed by the Find Peaks function (default parameters) in the Biomedical Image Analysis Research (BAR) plugin suite in Fiji. The distances between adjacent peaks were calculated and averaged to generate one value for each line. Two lines were drawn and analyzed per fiber, 5–6 fibers were analyzed per mouse, and 3 mice were analyzed for each antibody. The exact same fibers, easily identifiable in the serial, subsequent tissue sections, were analyzed for each antibody. The distances between peaks were plotted as paired sets (average distance as determined by N-terminal antibody; average distance as determined by C-terminal antibody) for each fiber analyzed.

#### Synthesis of peptides for mass spectrometry

Peptides were synthesized by the UNC Peptide Synthesis Core Facility at The University of North Carolina at Chapel Hill. Peptide 1 (KEEEQATETQPIVYATISASSASI) and peptide 2 (ALEHFTDLYDIK) were synthesized using Fmoc-Ile-Wang resin and Fmoc-Lys(Boc)-Wang resin, respectively, on a CEM Liberty Blue microwave peptide synthesizer using a modified Fmoc High-Efficiency Solid-Phase Peptide Synthesis (HE-SPPS) protocol as previously described.^87^ Following synthesis, the peptides were cleaved from the resin, and side-chain protecting groups were removed by treatment with a cleavage cocktail consisting of 95% trifluoroacetic acid, 2.5% water, and 2.5% triisopropylsilane for 2 h at room temperature. Crude peptides were precipitated by addition of the cleavage solution to cold diethyl ether, collected by centrifugation, and washed twice with cold diethyl ether. Subsequently, the crude peptides were dissolved in water, lyophilized, and purified by preparative reverse-phase high-performance LC (RP-HPLC). Purified peptides were then lyophilized and characterized by analytical RP-HPLC and matrix-assisted laser desorption/ionization mass spectrometry (MALDI-MS).

#### CLTC enrichment in mouse striated muscle samples by immunoprecipitation

Protein tissue lysate preparation from adult (P90) mice was performed and quantified as described above. Total protein (1 mg) for each sample was incubated with 3 μg of rabbit recombinant monoclonal IgG clathrin heavy chain antibody [EPR12235(B)] (epitope: short synthetic peptide within residues 200–300) (Abcam, ab172958) or 3 μg of a rabbit IgG isotype control recombinant antibody (Proteintech, #98136-1-RR) at 4°C overnight. The next day, protein samples were incubated with 1.5 mg of Dynabeads Protein G magnetic beads (Invitrogen, #10007D) for 2 h at 4°C. Following the incubation, the magnetic bead-antibody-antigen complexes were pelleted using a magnet and washed three times with RIPA lysis buffer (50 mM Tris-HCl pH 8.0, 150 mM NaCl, 5 mM EDTA pH 8.0, 0.1% SDS, 0.5% sodium deoxycholate, 1% NP-40). Samples used to confirm CLTC enrichment by western blotting were resuspended in 50 μL RIPA buffer supplemented with NuPAGE LDS buffer (Invitrogen, NP0007) and 50 mM dithiothreitol (Sigma-Aldrich, D0632-10G). Samples were incubated at 70°C for 10 min to dissociate the magnetic bead-antibody-antigen complex. Then, the magnetic beads were removed using a magnet and the immunoprecipitated lysate was assayed by western blotting as described above. The magnetic bead complex samples used for mass spectrometry were washed five times with distilled water using a magnet to pellet the magnetic bead-antibody-antigen complex between washes. Following the final wash, all remaining liquid was removed, and the magnetic beads were used for trypsinization and targeted mass spectrometry.

#### Targeted LC – mass spectrometry PRM (parallel reaction monitoring) assays

Following immunoprecipitation steps, samples were digested on beads with trypsin in 50 mM ammonium bicarbonate at 37°C overnight. Digested peptides were removed from the beads with two successive washes of water and acidified with trifluoroacetic acid to a final concentration of 0.2%. The peptides were desalted using C18 desalting columns (Thermo Fisher Scientific, #89852), lyophilized and resuspended in 2% acetonitrile with 0.1% formic acid for LC-MS/MS analysis. Peptide separation was performed using a Thermo Scientific UltiMate 3000 RSLCnano. Samples were separated on an IonOpticks Aurora Elite column (15 cm), which was heated to 50°C. The separation protocol used a 90 min gradient from 2 to 40% B with a flow rate of 250 nL/min (mobile phase A: 0.1% formic acid in water; mobile phase B: 0.1% formic acid in 80% acetonitrile).

Data acquisition was performed on a Thermo Scientific Orbitrap Exploris 480 mass spectrometer equipped with a Nanospray Flex ion source. The mass spectrometer was operated in positive ion mode with a static spray voltage of 2,100 V and an ion transfer tube temperature of 275°C. Precursor scans were acquired at 120 K resolution from 375–1500 m/z. The automatic gain control (AGC) target was set to 300% and the radio frequency (Rf) lens amplitude to 40% with a maximum injection time of 60 ms. Targeted MS2 (MS/MS or tandem mass spectrometry) scans were acquired for a predefined mass list over specific scheduled retention time windows (Table S2). Precursor ions were isolated using a 1 m/z isolation window and fragmented using HCD (higher-energy collisional dissociation) with a normalized collision energy of 30%. The MS2 scans were acquired in profile mode at a resolution of 60,000. The AGC target was set to Standard and maximum injection time of 120 ms utilizing 1 microscan. The Rf lens amplitude was customized to individual peptides. Data was processed using Skyline (64-bit) 26.1.^82^

#### Statistical analysis

The GraphPad Prism Software (version 10.1.2) was used for the statistical analysis of the data. Results are reported as the mean + the standard error of the mean (SEM). All experiments were performed using a minimum of three independent replicates, except those involving human fetal RNA samples where we only had access to two independent replicates. The statistical tests used for each experiment and the specific information about sample replicates are detailed in the relevant figure legends.
