# Supplementary material for: Alternative splicing of a species- and skeletal muscle-specific microexon diversifies the menu of clathrin heavy chains in mice and rats
Source: iScience. 2026 Oct 7;29(11):117799. doi: 10.1016/j.isci.2026.117799 (PMC13676984; doi:10.1016/j.isci.2026.117799)
Supplement: Supplementary file 1 [file mmc1.pdf]

iScience, Volume *29*

Supplemental information

Alternative splicing of a species- and skeletal muscle-specific microexon diversifies the menu of clathrin heavy chains in mice and rats

R. Eric Blue, Adam J. Black, Jessica L. Cote, Sumaya A. Addish, Archishma Kavalipati, Scott P. Lyons, J. Ashley Ezzell, Steven C. Bremmer, Daniel Dominguez, and Jimena Giudice

SUPPLEMENTAL FIGURES AND LEGENDS

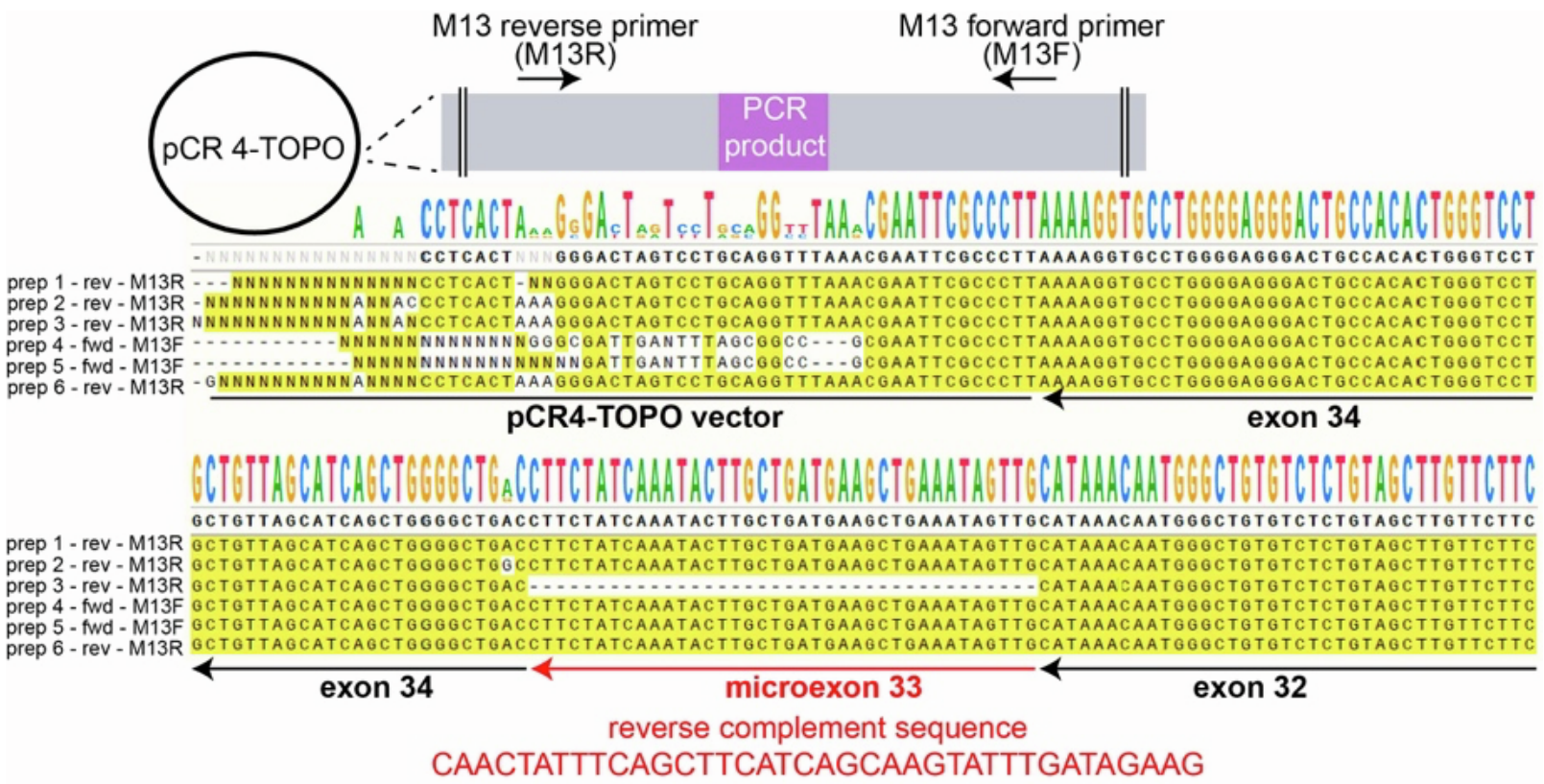

Figure S1. TOPO cloning Cltc microexon 33 confirms its sequence and size. Related to Figure 1A.

PCR amplified cDNA from an adult (postnatal day 120, P120) FVB/N mouse gastrocnemius muscle was cloned into the pCR 4-TOPO vector and transformed into competent *E. coli* DH5α cells that were further cultured in agar plates. Six single colonies were selected, separately cultured, and used to purify plasmid DNA that was then analyzed by Sanger sequencing using the universal M13 reverse (M13R) and M13 forward (M13F) primers (black arrows, top). In five out of six colony preparations (prep), the expected sequence of Cltc microexon 33 (CAA-CTA-TTT-CAG-CTT-CAT-CAG-CAA-GTA-TTT-GAT-AGA-AG) flanked by exons 32 and 34 and its size (38 nt) were confirmed. **Figure S1** relates to **Figure 1A**.

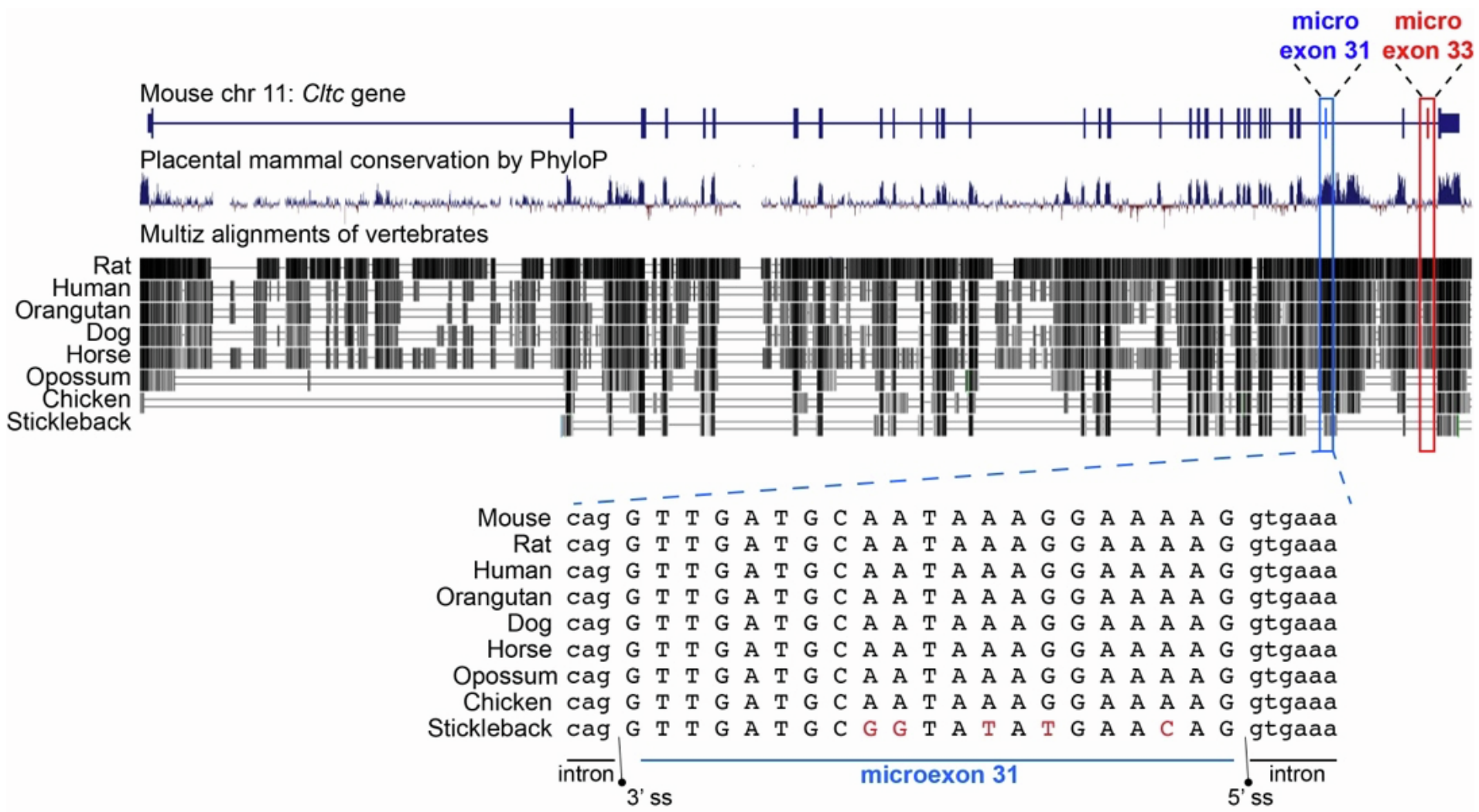

Figure S2. Cltc microexon 31 is highly evolutionarily conserved. Related to Figure 1C.

Plot of basewise evolutionary conservation of the *Cltc* gene as determined by PhyloP score and visualized in the UCSC genome browser. Alignments of different vertebrate species were obtained from the UCSC genome browser which generated them with multiz and other tools in the UCSC/Penn State Bioinformatics comparative genomics alignment pipeline. Bases that differ from those in the mouse sequence are shown in red. chr: chromosome, ss: splice site. **Figure S2** relates to **Figure 1C**.

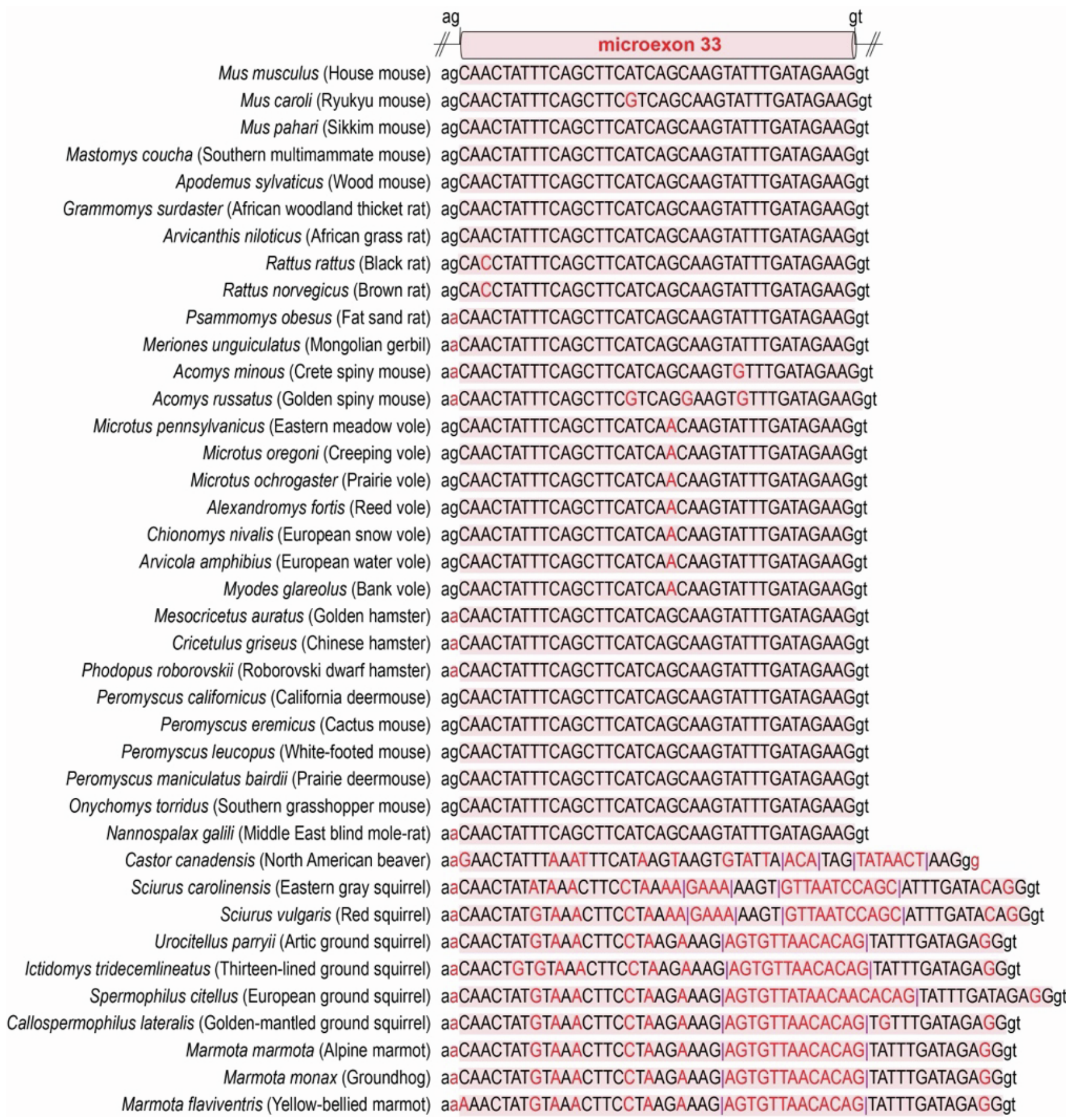

Figure S3. Rodent sequence alignment of the mouse Cltc microexon 33 shows the highest sequence homology between mouse, rat, gerbil, vole, hamster, and deermouse species. Related to Figure 1D.

Sequence alignment was performed using the BLAST nucleotide search tool from the NCBI. The mouse intronic DNA sequence between Cltc exons 32 and 34 was used as the source input and compared with the available genomes in the *rodentia* order. Species listed exhibited the highest alignment scores. The displayed sequences denote the individual nucleotides present in microexon 33 and its splice sites in the different rodent species. Bases that differ from the mouse sequence are shown in red. The purple brackets denote a sequence insertion. **Figure S3** relates to **Figure 1D**.

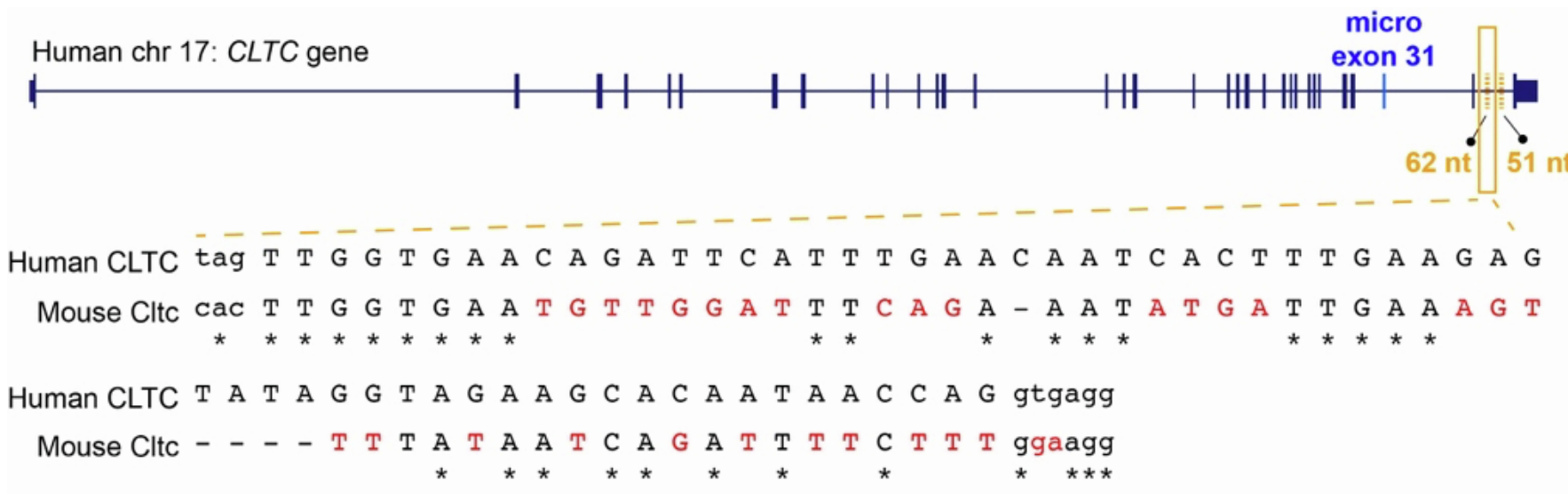

Figure S4. A 62 nt-long sequence in the human CLTC intron 32. Related to Figure 1D.

Human *CLTC* gene structure. Inspection of human *CLTC* intron downstream of exon 32 revealed that 1,230 nt upstream the 51 nt-long sequence highly homologous to the mouse Cltc microexon 33 (except the insert) (see **Figure 1C-D**), there was a 62 nt-long sequence flanked by a more canonical 3' splice site (tag) that does not align well with the mouse Cltc sequence. Although the 62 nt-long sequence appears to be annotated in some human transcript assemblies, no information about the existence of the potential transcripts has been found in the databases. Mouse bases that differ from those in the human sequence are shown in red. * indicate bases that are the same in the human and mouse genome. chr: chromosome. **Figure S4** relates to **Figure 1D**.

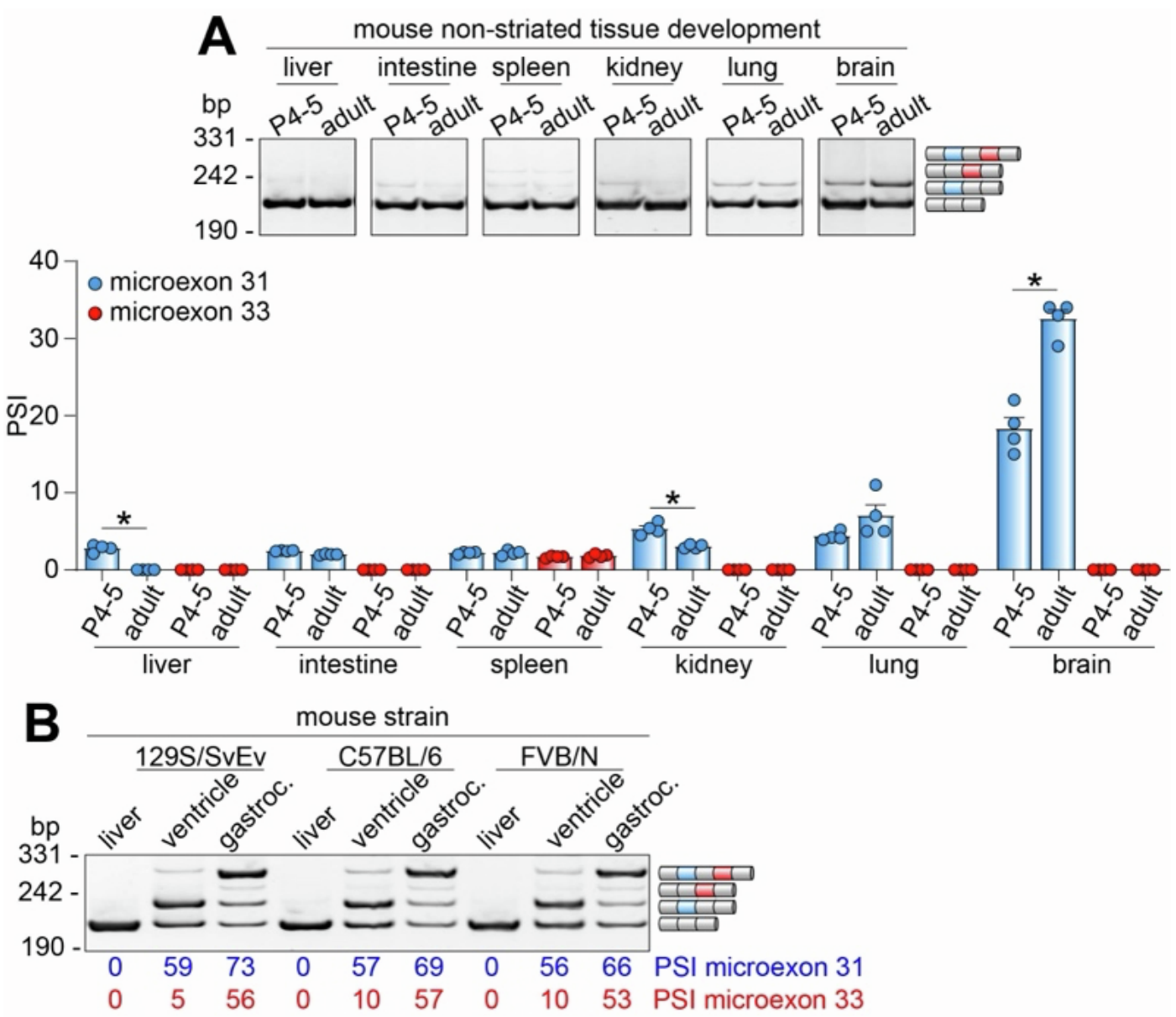

Figure S5. The splicing profiles of Cltc microexons 31 and 33 differ between mouse striated muscles and other tissues. Related to Figure 2.

(A) Alternative splicing of Cltc microexons 31 and 33 was evaluated by reverse transcription PCR (RT-PCR) assays followed by polyacrylamide electrophoresis in mouse neonatal (postnatal days 4 and 5, P4-5) and adult (4 months of age) liver, intestine, spleen, kidney, lung, and brain tissues. The level of exon inclusion (percent spliced in, PSI) was evaluated by densitometry. Results are shown as mean + SEM. *$p<0.05$, two-way ANOVA with Tukey's post-hoc multiple comparison test, n=4 biological replicates.

(B) Alternative splicing of Cltc microexons 31 and 33 was evaluated by RT-PCR assays followed by polyacrylamide electrophoresis in liver, ventricle, and gastrocnemius (gastroc.) muscle tissues of commonly used mouse strains (129S/SvEv, C57BL/6, and FVB/N) in adulthood (4-5 months of age). The PSI was evaluated by densitometry. bp, base pairs.

Figure S5 relates to **Figure 2**.

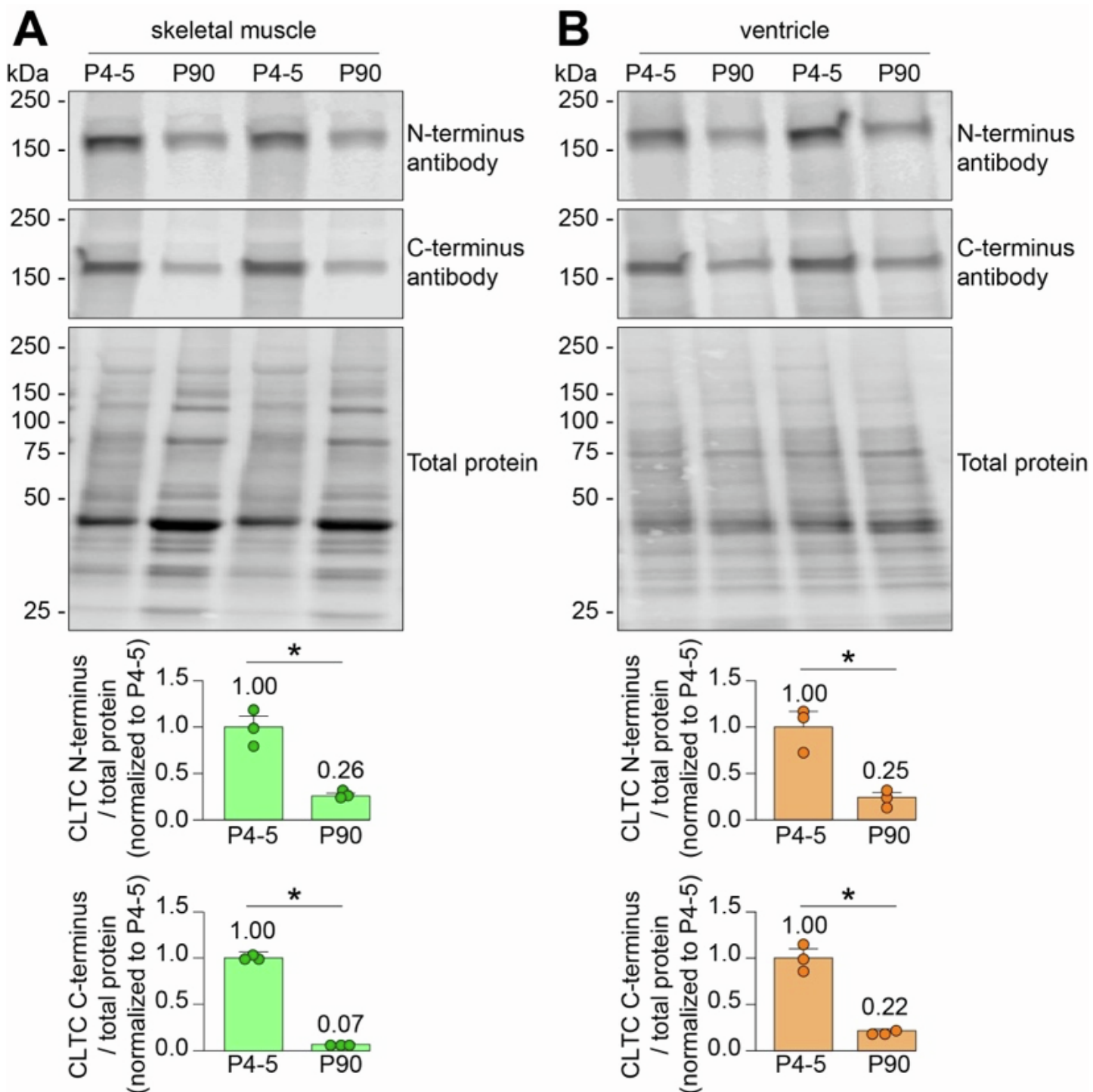

Figure S6. CLTC is downregulated during striated muscle development. Related to Figure 5.

(A-B) Antibodies targeting the clathrin heavy chain N- or C-termini were used to evaluate CLTC protein levels in gastrocnemius muscles **(A)** and ventricles (**B**) from neonatal (postnatal days 4 and 5, P4-5) and adult (P90) mice via western blotting. Quantifications were performed by densitometry. Results are shown as mean + SEM. *p<0.05, unpaired Student's t-test, n=3 biological replicates. **Figure S6** relates to **Figure 5**.

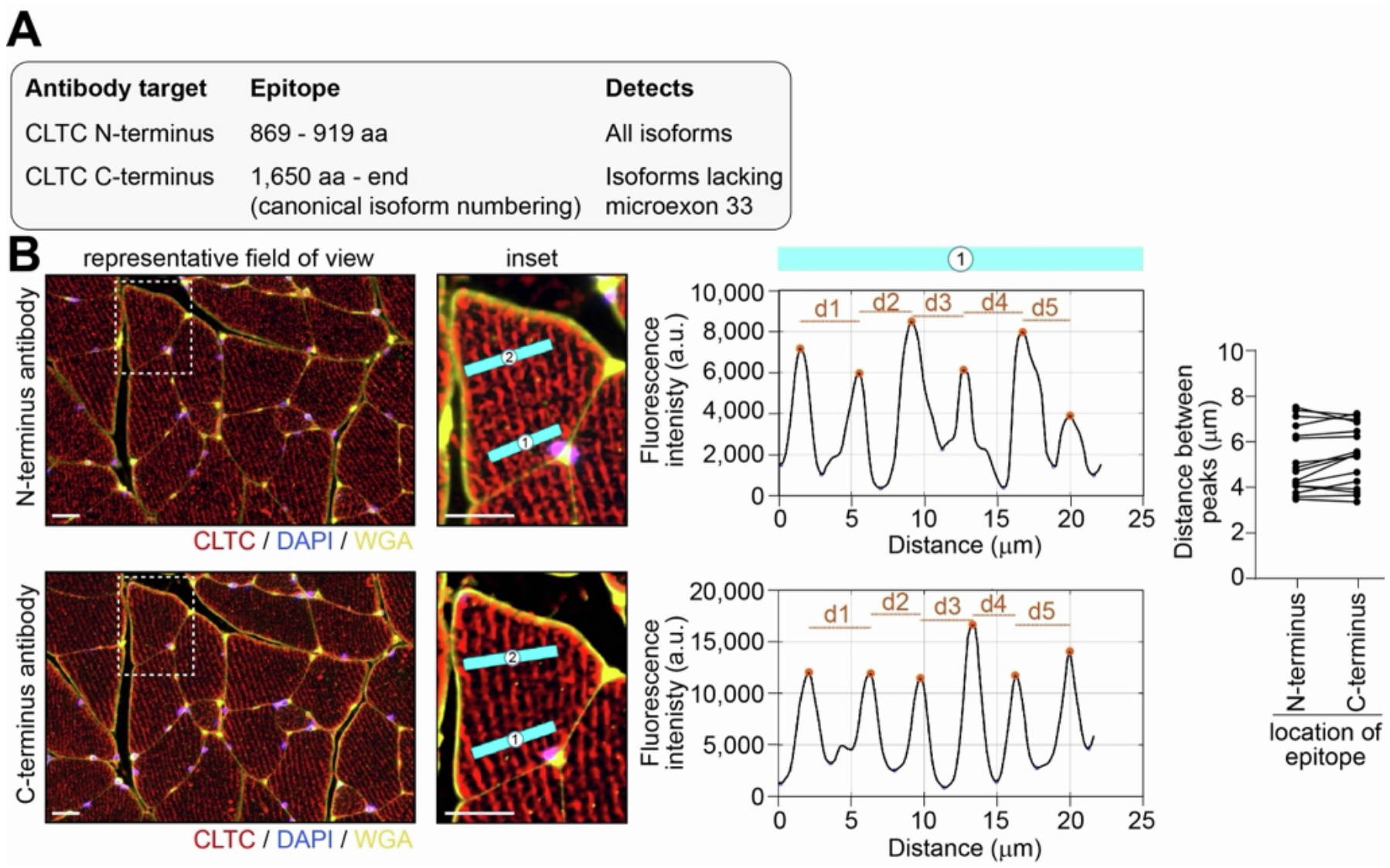

Figure S7. CLTC is primarily arranged in a striated pattern within fiber cross sections. Related to Figure 5.

(A) Expression patterns of CLTC protein in gastrocnemius muscle tissue sections of adult mice were obtained by immunofluorescence using antibodies targeting its N- or C-termini.

(B) Serial transverse sections from gastrocnemius muscles of adult C57BL/6 mice were immunostained with an anti-clathrin heavy chain antibody targeting the N-terminus (top) or the C-terminus (bottom) of the protein and a secondary antibody conjugated to Alexa Fluor Plus 647 (red). The plasma membrane of the muscle fibers was stained with wheat germ agglutinin (WGA) conjugated to Alexa Fluor Plus 568 (yellow) and nuclei with DAPI (blue). In the images (left panels show representative ones), the background was subtracted from the red channel only. Dashed white rectangles: areas shown at higher magnification in insets (middle panels). Scale bars: 20 μm. Inset images were processed for quantitative analysis including background subtraction, median filters, local contrast enhancement, and line scans, all of which were applied to the red channel only. In each analyzed myofiber, two lines were drawn (cyan lines 1 and 2 in insets) and used to generate fluorescence intensity plots (middle panels; orange circles: maximum intensity of the peaks; orange horizontal dotted lines (d_i): distances between adjacent peaks). For each analyzed myofiber, a mean distance between peaks was calculated for line 1 and for line 2, and then these two values were averaged (right panel). The distances between peaks were not significantly different between the two antibodies by paired Student's t-test (n=16 total fibers per antibody, from 3 mice with 5-6 fibers per mouse).

Figure S7 relates to **Figure 5**.

SUPPLEMENTAL TABLES

Gene symbol	Species	Alternative exon size (bp)	Primer direction	Primer sequence	Expected sizes (bp) of PCR bands
Cltc	*Mus musculus*	38	forward	CGGAGTCGCTGAGAAAAGAAG	157, 119
			reverse	AAAAGGTGCCTGGGGAGG	
Cltc	*Rattus norvegicus*	38	forward	CAGAGACACAGCCCATTGTTT	187, 149
			reverse	TCACATGCTGTACCCAAAGC	
CLTC	*Gallus gallus*	not expected*	forward	TGGATGCATCAGAGTCCTTGA	157
			reverse	CCGTATGGTGGTGCAGTGTA	
CLTC	*Homo sapiens*	51 or 62	forward	AGCTACAGAGACACAACCCA	209, 198, 147
			reverse	CTGTACCCAAAGCCAGGC	
CLTCL1	*Homo sapiens*	40	forward	GCAAGAGGAGCATGTGACAG	171, 131
			reverse	ACGCGGAAGTTGTACATTGG	
Cltc	*Mus musculus*	21, 38	forward	GAAACCGCATGGAGACATAA	275, 254, 237, 216
			reverse	AAAAGGTGCCTGGGGAGG	
Cltc	*Rattus norvegicus*	21, 38	forward	GAAACTGCATGGAGACATAA	338, 317, 300, 279
			reverse	TCACATGCTGTACCCAAAGC	
Srsf3	*Mus musculus*	456	forward	TGGAACTGTCGAATGGTGAA	589, 133
			reverse	GAAAAGCTTCTCCTTCTTGG	

Table S1. Alternative splicing primers. Related to STAR Methods.

Sequence and detailed information about the primers (Sigma-Aldrich) used for alternative splicing analysis. The alternative exon size was calculated from sequence alignment of the species. *denotes the 38 nt sequence of microexon 33 that is absent in the *Gallus gallus* intronic sequence between the flanking exons 32 and 34. bp: base pairs.

Peptide sequence	m/z	z	Start time (min)	End time (min)	HDC Collison energies	Rf lens
KEEEQATETQPIVYATISASSASI	1,277.1320	2	40	50	25%	50%
ALEHFTDLYDIK	732.8721	2	36	40	30%	40%

Table S2. Targeted peptide inclusion list for targeted mass spectrometry study. Related to STAR Methods.

Amino acid sequence and features of the targeted peptides: precursor peptide mass-to-charge ratio (m/z) for the selected ion, charge state of the monitored precursor ion (z), scheduled retention time to begin and end acquisition of data for the precursor ion (start time and end time, respectively), specific collision energy utilized for the fragmentation of the targeted precursor ion (HCD Collision energies), and specific radio frequency amplitude (Rf lens) utilized for transmission of the targeted precursor ion.
